# Discovery of selective monosaccharide receptors *via* dynamic combinatorial chemistry†

**DOI:** 10.1039/d4ob00015c

**Published:** 2024-04-12

**Authors:** Miguel Alena-Rodriguez, Marcos Fernandez-Villamarin, Ignacio Alfonso, Paula M. Mendes

**Affiliations:** a School of Chemical Engineering, University of Birmingham Edgbaston Birmingham West Midlands B15 2TT UK p.m.mendes@bham.ac.uk; b Department of Biological Chemistry, Institute for Advanced Chemistry of Catalonia, IQAC-CSIC Jordi Girona 18-26 08034 Barcelona Spain

## Abstract

The molecular recognition of saccharides by synthetic hosts has become an appealing but elusive task in the last decades. Herein, we combine Dynamic Combinatorial Chemistry (DCC) for the rapid self-assembly and screening of virtual libraries of receptors, with the use of ITC and NMR to validate the hits and molecular modelling to understand the binding mechanisms. We discovered a minimalistic receptor, 1F (*N*-benzyl-l-phenylalanine), with considerable affinity for fructose (*K*_a_ = 1762 M^−1^) and remarkable selectivity (>50-fold) over other common monosaccharides. The approach accelerates the discovery process of receptors for saccharides.

## Abstract 2

An effective workflow to discover selective saccharide receptors by combining dynamic combinatorial chemistry with isothermal titration calorimetry and NMR.

## Graphical abstract

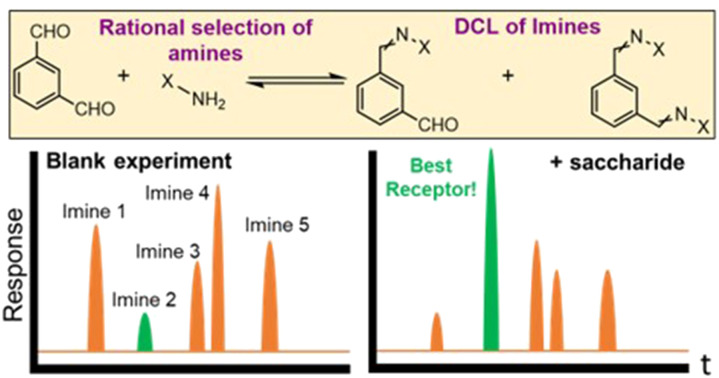

## Introduction

The precise recognition of saccharides, more commonly known as carbohydrates or sugars, has gained significant relevance in the past years due to their key role in the appropriate functioning of organisms. Alteration of their normal physiological levels, whereas they are free or conjugated with more complex biomolecules, is associated with the development of diseases such as diabetes, Crohn's disease, or cancer.^1–3^ Therefore, the recognition of saccharides by artificial hosts represents an excellent approach for the molecular study of these diseases.

Despite the apparent simplicity of sugars, these molecules are a challenging target in biological samples. The three most common monosaccharides are glucose, fructose, and galactose, which are isomers with a 6-carbon backbone and different arrangements of their functional groups. The similarity among sugars, together with the fact that they are normally heavily hydrated in aqueous environments,^4,5^ has made the selective recognition of carbohydrates a challenging task that has attracted researchers for the last 35 years.^6,7^ From the first sugar-receptor ever designed by Aoyama in 1988,^8^ with discrete affinities and selectivities for their target, and unable to work in aqueous media to probably the most powerful sugar receptor synthesised yet, developed by Davis in 2019,^9^ the field of molecular recognition of monosaccharides have witnessed enormous progression. However, these advances were mostly based on rational design and ultimately, proof and error of individual candidates.

**Fig. 1:**
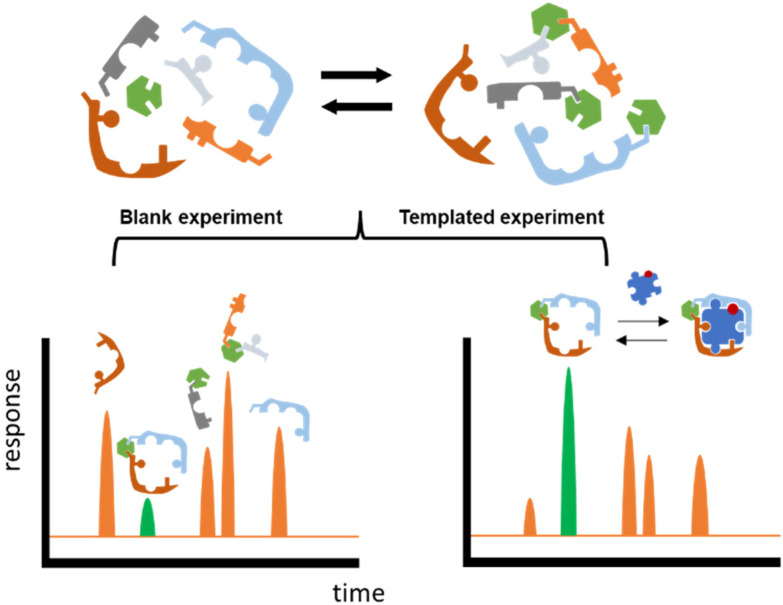
Schematic representation of DCC protocol. The figures on top represent the starting building blocks in the Dynamic Combinatorial Library (DCL) reversibly reacting with one another. In the bottom, a screening of the DCLs in absence (blank experiment) and in presence (templated experiment) of the saccharide template (represented as a puzzle piece). The library member with the highest affinity for the template gets amplified in the templated experiment (highlighted in green).

Herein we propose an approach that could potentially accelerate the discovery process. The core of this methodology is the well-known technique named Dynamic Combinatorial Chemistry (DCC).^10–14^ DCC relies on the dynamic generation of interconvertible species *via* reversible reactions between starting building blocks (BBs), and the ability of this system to respond to external stimuli such as a change in temperature, pH, or the addition of an external molecule (template). This last situation will be exploited in this work, the template being a carbohydrate that will trigger the self-assembly and system-enrichment of the best possible hosts with the highest affinity for a given sugar template (Fig. 1).

DCC has been thoroughly implemented in the successful discovery of receptors for a number of biomolecules: peptides,^15^ DNA,^16^ RNA,^17^ proteins,^18^ complex glycosaminoglycans^19^ and even whole living cells.^20^ Nevertheless, this technique has been far less explored in the discovery of receptors for monosaccharides,^21^ perhaps due to their aforementioned difficulties. In this work, we present a novel workflow that combines the rational selection of BBs to perform DCC experiments with the individual synthesis and validation by ITC and NMR of the most promising candidates. The knowledge obtained from these analyses with regards to the most relevant features to achieve binding can then be implemented in the re-design of further optimised Dynamic Combinatorial Libraries (DCLs) that will produce even better fitting receptors.

## Results and discussion

**Fig. 2:**
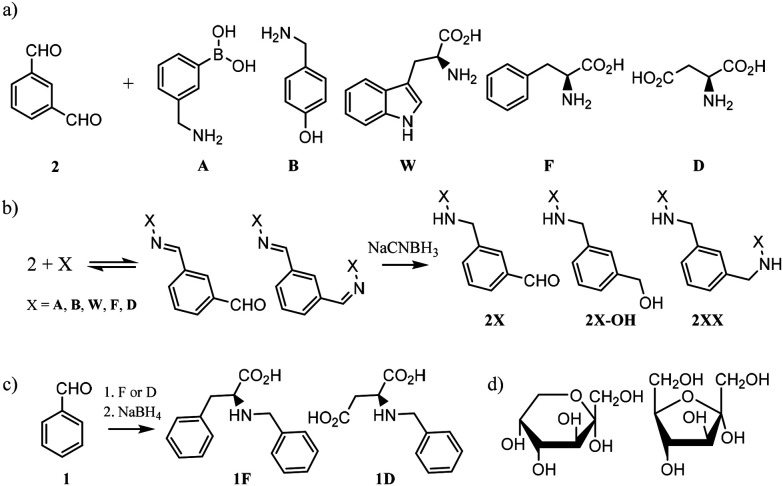
(a) Building blocks selected for our DCC experiments. (b) Imine formation reaction in equilibrium followed by irreversible reduction with NaCNBH_3_. The non formation of 2X-OH was confirmed by LC-MS. (c) Protocol for the syntheses of 1F and 1D. (d) Representation of the pyranose (left) and furanose (right) forms of monosaccharides in solution. α-D fructose drawn as an example.

In order to design the DCL we considered BBs that meet three crucial requirements: (i) possess functional groups capable of reversible exchange, (ii) contain groups potentially able to interact with the templates, and (iii) can offer multivalency, as this is known to enhance binding affinities. In terms of the geometrical shape of the desired receptors, there are a few designs that have proven to be effective in the past for the recognition of sugars. From 3D cage-like structures,^9,22^ to linear oligomers that fold to encapsulate their target.^23^ However, we pursued a more simple design that could be afforded with inexpensive and readily available starting materials. With this in mind, we selected the BBs shown in Fig. 2a. Isophthalaldehyde (2) as the central scaffold would ensure reversibility and multivalent library members upon imine formation reaction with BBs A, B, W, F, and D. This set of molecules would enrich the library with an array of functionalities capable of creating interactions with the target saccharides. In particular, boronic acids (A) are known to bind diols,^24^ extensively present in saccharides. Phenylalanine (F) and aspartic acid (D) were recently reported to have excellent binding properties for sugar-containing glycans.^25^ Furthermore, tyrosine (represented by 4-(aminomethyl)phenol, B) and tryptophan (W) are also known to be extensively present in binding pockets of carbohydrate-recognition lectins.^26^ In summary, the BBs selected ensured a DCL rich in functionalities able to participate in both covalent and non-covalent interactions with the target saccharides. Particularly, we promoted the possibility of CH–π interactions as these have proven to drive molecular recognition processes with glycans and carbohydrates.^27,28^

Since imines are not stable in the conditions required for the analysis of the DCLs,^29^ we reduced them with NaCNBH_3_ prior analysis. This reduction step is irreversible and therefore the equilibrium composition was frozen, as shown in Fig. 2b. NaCNBH_3_ was chosen as it is a mild reducing agent that effectively reduces imines to their corresponding secondary amines (2X, Fig. 2b).^30^ The products of reduction of the aldehyde groups in 2X (2X-OH, Fig. 2b) were not detected by LC-MS in any DCC experiment (ESI†). With these BBs and the conditions previously optimised by us, we performed different DCC experiments employing four isomeric sugars as templates: d-glucose, d-mannose, d-galactose and d-fructose. Each experiment consisted of a positive and a negative experiment, performed as detailed in ESI.† The DCLs were analysed by LC-MS and library members were detected and quantified according to the corresponding (M + H)^+^ ions formed in the extracted ion chromatogram (EIC). The potentially competing reductive amination reaction between the amine building blocks and the open forms of the saccharides was ruled out since the corresponding by-products were not detected by LC-MS in any of the assayed libraries.

Amplification (*A*) values were calculated as the division between the LC-MS peak intensity (in the EIC) in presence and in absence of the template, meaning that values *A* > 1 correspond to actual amplification of the species in the presence of the template while values *A* < 1 means the opposite. This methodology therefore infers that *A* values are inevitably related to the binding constant of a library member and a template, but it is worth highlighting that when comparing different DCC experiments, *A* value is rather a function of selectivity and not of affinity for the template of study in each experiment.^31^ In other words, *A*_2DD__-glucose_ = 3.43 means that 2DD should have a better binding affinity (higher *K*_a_) than any other virtual DCC member with *A* < 3.43 in the experiment with glucose as template. However, *A*_1F__-fructose_ = 2.11 could correspond (and in fact it does, see below) with a higher *K*_a_ value for the corresponding 1F-fructose complex.

**Fig. 3:**
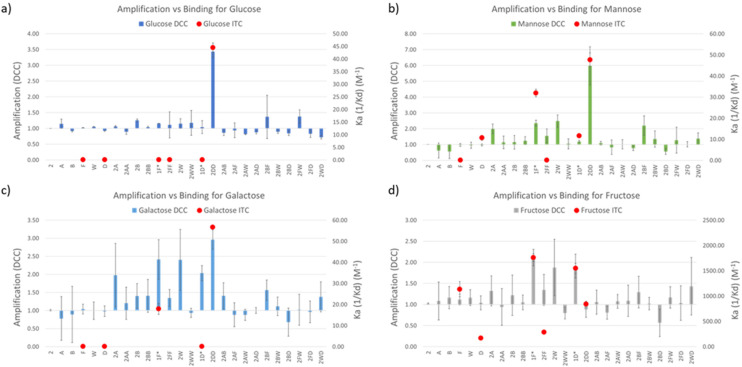
DCC results (amplification, left *Y* axes, coloured bars) and ITC results (*K*_a_, right *Y* axes, red dots) plotted together for the experiments with (a) glucose, (b) mannose, (c) galactose, and (d) fructose. *The molecules tested by ITC were 1F and 1D but the plotted ‘*A*’ values in those positions in the bar graph were those of 2F and 2D, respectively.

DCC experiments were performed in triplicate and the average *A* values, plus the standard deviation, are plotted in Fig. 3a–d (left *Y* axes, coloured bars). While both the net *A* values and their deviations varied quite drastically across the different experiments, the trend remained the same in experiments with glucose, mannose, and galactose, with library member 2DD standing out as the best receptor in all the three cases. The experiment with fructose resulted in a much different output with 2D and 2F as the most promising receptors in a more competitive environment. In order to correlate *A* values with affinity for the template, library members 2D, 2F, 2DD, and 2FF were individually synthesised by reductive amination, and they were tested by ITC to find their binding constants towards the saccharides of interest.

Commercially available amino acids D and F were assayed too. As the main objective of this research was to create a simple methodology for the rapid screening and testing of hits, we decided to simplify the synthesis of monosubstituted products 2D and 2F. Benzaldehyde (1), instead of isophthalaldehyde (2), was employed (Fig. 2c) thus avoiding the extra synthetic steps or tedious purification protocols derived from the presence of an extra reactive aldehyde in the starting material. Hence, the molecules tested by ITC were 1D and 1F, differing from the actual library members in the DCC experiment in the lack of a CHO group in *meta* in the central aromatic ring. However, that group being distant from the potential recognition region of the molecule, we believe that its absence should not affect the overall binding properties of the receptors. Notably, ITC and DCC results were in good agreement which suggests that this substitution did not endanger the usability of the protocol (Fig. 3).

In the experiments with glucose, mannose, and galactose as templates 2DD was the best receptor, as predicted by DCC. *K*_a_ values were in the three cases in the range of 45 M^−1^ (Table 1). The similar behaviour of the three monosaccharides did not come as a surprise since they are isomeric structures, differing only in the spatial orientation of their hydroxyl groups. More interesting results were found in the experiment with fructose. The dissimilarity observed by DCC between fructose and the other sugars was supported by ITC data, with the highest *K*_a_ values being significantly higher than for the other saccharides. Remarkably, *K*_a__1D__-fructose_ and *K*_a__1F__-fructose_ were 1548 ± 98 and 1762 ± 162 M^−1^, respectively. To the best of our knowledge, the binding constant exhibited by 1F for d-fructose (1762 M^−1^) is among the highest yet reported by synthetic small molecules for such saccharide in water environments. It surpasses reported values of phenylboronic acid (PBA, *K*_a_ = 560 M^−1^) or *o*-amino PBA (*K*_a_ = 1640 M^−1^),^32^ both relying on covalent bond formation with the sugar. However, there are examples of receptors of this type that outperformed 1F, for instance, the bis-boronic acid reported by Fossey *et al.* that binds d-fructose with *K*_a_ = 130 × 10^3^ M^−1^.^33^

**Table 1:** Affinity values (*K*_a_, M^−1^), measured by ITC, of the set of molecules and sugars tested, and selectivity of molecule 1F for fructose calculated by dividing the affinity of 1F for fructose over its affinity for the other saccharides tested

	d-Glucose	d-Galactose	d-Mannose	d-Fructose
D	0.0	0.0	10.7 ± 1.2	170.7 ± 39.6
F	0.0	0.0	0.0	1136.0 ± 148.3
1D	0.0	0.0	11.7 ± 0.7	1548.3 ± 98.1
1F	0.0	17.9 ± 2.6	31.9 ± 2.1	**1762.0 ± 162.4**
2DD	44.5 ± 2.0	56.7 ± 0.3	47.7 ± 3.5	840.7 ± 39.0
2FF	0.0	0.0	0.0	289.0 ± 21.0

Selectivity	>100	98.4	55.2	1.0

Fructose is an isomer to the other tested sugars, but it is the only ketose of the series, which produces a considerably higher amount of its five-membered furanose forms in comparison with the other saccharides tested (28.6% *vs.* 0.1–6%)^34,35^ and a different structural arrangement around the anomeric carbon (see furanose and pyranose forms of d-fructose in Fig. 2d). It was also interesting to find that 1D and 1F show better binding affinity than their bidentate analogues 2DD and 2FF (and better than D and F alone), suggesting that our DCC approach led to the optimal size for the receptors. The ITC titration graph of molecules 1D and 1F with d-fructose also revealed that the binding is mainly entropy driven (Δ*S* dominates Δ*G*, ESI†). For molecules like these ones that are small and relatively flexible, it can be hypothesized that this relatively strong binding may be attributed to desolvation processes. Another interesting and unexpected outcome was the positive behaviour of 2DD in the experiments with glucose, mannose, and galactose. Posing only 1 aromatic unit, 2DD outperformed other competitors with greater ability to offer CH–π interactions. It could be proposed that H bond formation was the preferential intermolecular force between 2DD and such sugars. In terms of selectivity, the binding affinity of 1F for fructose was 55 times higher than for mannose and 98 times higher than for galactose (Table 1). Its affinity for glucose was too weak to be accurately measured with our ITC equipment, which suggests that the selectivity of 1F for fructose over glucose should be even higher (>100-fold) than over galactose.

**Fig. 4:**
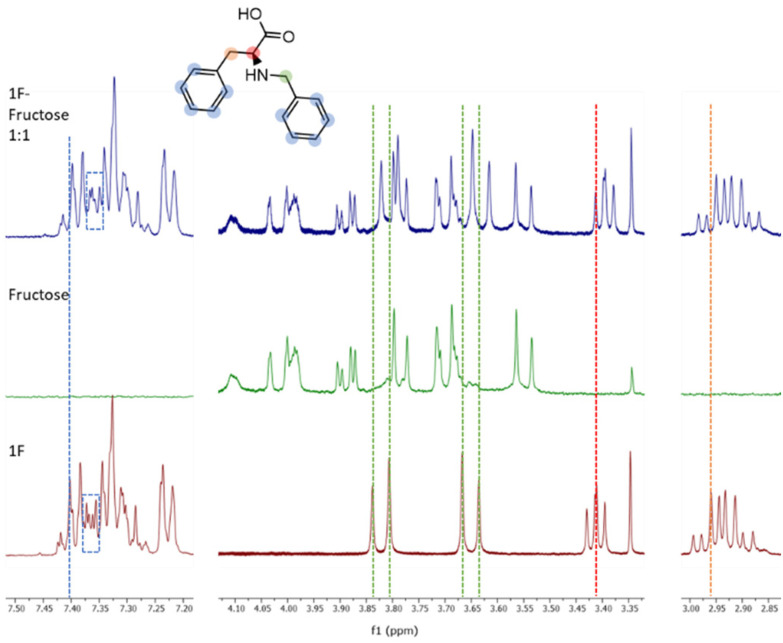
Stacked ^1^H NMR spectra (400 MHz, carbonate buffer in D_2_O, 298 K) of receptor 1F (bottom), fructose (middle) and the equimolar mixture of 1F and fructose (top). The structure of the receptor 1F is drawn and the shift of the signals can be visualised with the coloured dotted lines.

Molecule 1F was further analysed by NMR titrations to understand the mechanisms behind the binding phenomena. From the binding constants obtained from ITC, we estimated that the optimal concentrations of receptor and sugar to maximize the formation of 1F-fructose complex was 10 mM for both. Unfortunately, due to low solubility of 1F, titrations were performed at lower concentrations, which limits the applicability of the results. 1F was kept constant at 0.9 mM and different concentrations of fructose (below, above, and at 0.9 mM) were tested. The maximum displacements of chemical shifts were observed at equimolar concentrations. Under these conditions all the signals in 1F shifted upfield, although they did it to different extents. Aromatic protons (7.45–7.20 ppm) experienced very small change (Fig. 4, blue dotted line). It was also appreciated a subtle modification in the shape of some of those peaks (Fig. 4, dotted rectangles) although this region of the spectrum is too complex to be analysed in detail. Both groups of protons next to the secondary amine (*i.e.*, benzylic, and alpha amino acid protons, green and red lines in Fig. 4, respectively) suffered the largest displacements (Δ*δ* = 0.02 ppm). Finally, the beta protons of amino acid (orange line in Fig. 4) shifted Δ*δ* = 0.008 ppm. The peaks of fructose did not shift at all. These results identify the most perturbed central moiety of 1F as key for the binding. The combined implication of the aromatic residues should not be disregarded though, especially considering that, as outlined before, the molecule with two aromatic rings (1F) outperforms those with three and one of them (2FF and F).

**Fig. 5:**
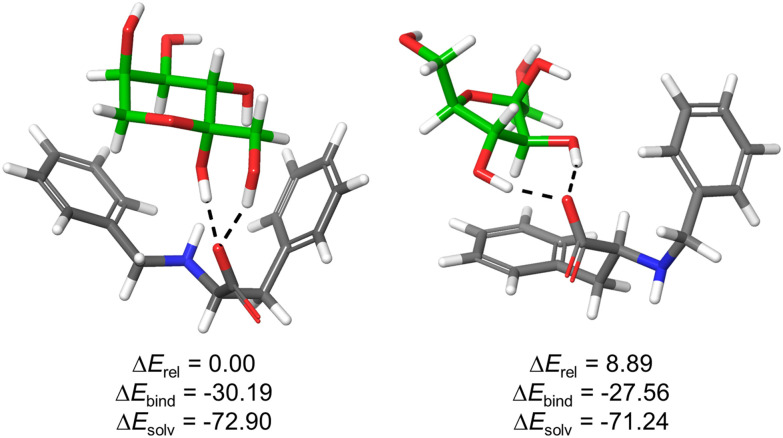
Optimized structures for the complexes formed between 1F and β-fructopyranose (left) or β-fructofuranose (right) collectively accounting for >90% of sugar isomers in water. Monosaccharides are displayed with green C-atoms and H-bonds are depicted by black dashed lines. Computed relative overall energies (Δ*E*_rel_), binding energies (Δ*E*_bind_ = *E*_complex_ − (*E*_1F_ + *E*_sugar_)) as well as solvation energies (Δ*E*_solv_ = *E*_water_ − *E*_gas_) of the corresponding complexes are given in kcal mol^−1^. For other representative optimized structures, see ESI.†

Considering the limited information obtained from the NMR titration experiments, we undertook molecular modelling studies on the intriguing [1F-fructose] complex. To this aim, we used the anionic carboxylate form of 1F and different isomers of d-fructose. We hypothesized that 1F is able to bind native forms of fructose according to the minor changes observed in the saccharide ^1^H NMR signals upon titration with 1F. Thus, we carried out Monte Carlo conformational searches with OPLS4 force field minimizations in implicit water followed by DFT geometry optimizations with water PCM solvation model (Fig. 5, see ESI† for details).

The results support the favourable formation of stable supramolecular complexes, as reflected in the negative values of Δ*E*_bind_. The structures are stabilized by hydrogen bonding interactions between sugar OH groups and 1F-carboxylate, in addition to potential C–H⋯π contacts between the substrate and the aromatic rings of the host. In the case of the five-member ring, the phenylalanine side chain is mainly implicated while for the pyranose form (most abundant isomer and most stable complex), both aromatic rings are pointing towards the sugar (Fig. 5). This defines a tweezer-like arrangement of carboxylate and aromatic rings that would efficiently de-solvate both host and guest, explaining the entropically-driven binding obtained experimentally by ITC. Actually, the highly negative solvation energies (Δ*E*_solv_) reflect the key role of the aqueous medium for the stabilization of the structures. Moreover, the most stable complex bears concerted H-bonds between carboxylate of 1F and both hemiacetal–OH and C1–OH of β-fructopyranose, suggesting a potential explanation for the observed ketose selectivity over the aldoses where this specific arrangement is not possible.

## Conclusions

In summary, this study provides a robust DCC methodology for the rapid discovery of receptors for the most common monosaccharides in Nature. Library member 2DD -two units of aspartic acid connected in *meta* to a benzene ring- was found to be the best receptor among the library members for d-glucose, d-mannose, and d-galactose, though with low selectivity among sugars. The d-fructose-templated DCC experiments identified molecule 1F (*N*-benzyl-l-phenylalanine), as the best host, with a considerably large binding constant (*K*_a_ = 1762 M^−1^) and selectivity (>50-fold) over the other saccharides tested. These are remarkable results considering that all tested sugars are isomers. Virtually any type of saccharide could be studied with this methodology. Likewise, the complexity and size of the DCL can be extended up to their analytical limitations, making this approach easily scalable with the potential to discover new selective saccharide receptors.

## Author contributions

M. A. conducted the experimental work, P. M., I. A. and M. F.-V. reviewed the manuscript.

## Conflicts of interest

There are no conflicts to declare.

## Supplementary Material

OB-022-D4OB00015C-s001

## References

[cit1] Bruen D., Delaney C., Florea L., Diamond D. (2017). Sensors.

[cit2] Clerc F. (2018). et al.. Gastroenterology.

[cit3] Munkley J., Elliott D. J. (2016). Oncotarget.

[cit4] Oshovsky G. V., Reinhoudt D. N., Verboom W. (2007). Angew. Chem., Int. Ed..

[cit5] Davis A. (2010). Nature.

[cit6] James T. D. , Phillips M. D. and Shink S., The Molecular Recognition of Saccharides, 2006

[cit7] Davis A. P. (2020). Chem. Soc. Rev..

[cit8] Aoyama Y., Yasutaka T., Hiroo T., Ogoshi H. (1988). J. Am. Chem. Soc..

[cit9] Tromans R. A. (2019). et al.. Nat. Chem..

[cit10] Lehn J. M. (1999). Chem. – Eur. J..

[cit11] Lehn J.-M., Eliseev A. V. (2001). Science.

[cit12] Cougnon F. B. L., Sanders J. K. M. (2012). Acc. Chem. Res..

[cit13] Mondal M., Hirsch A. K. H. (2015). Chem. Soc. Rev..

[cit14] Frei P., Hevey R., Ernst B. (2019). Chem. – Eur. J..

[cit15] Nicolaou K. C. (2001). et al.. Chem. – Eur. J..

[cit16] Whitney A. M., Ladame S., Balasubramanian S. (2004). Angew. Chem., Int. Ed..

[cit17] Bugaut A., Toulmé J. J., Rayner B. (2006). Org. Biomol. Chem..

[cit18] Monjas L., Hirsch A. K. (2015). Future Med. Chem..

[cit19] Carbajo D. (2022). et al.. J. Med. Chem..

[cit20] Carbajo D., Perez Y., Bujons J., Alfonso I. (2020). Angew. Chem., Int. Ed..

[cit21] Rauschenberg M., Bomke S., Karst U., Ravoo B. J. (2010). Angew. Chem., Int. Ed..

[cit22] Zhai C. (2023). et al.. Chem. – Eur. J..

[cit23] Chandramouli N. (2015). et al.. Nat. Chem..

[cit24] Wu X. (2013). et al.. Chem. Soc. Rev..

[cit25] Chen Z. (2018). et al.. NPG Asia Mater..

[cit26] Siebert H. C. (1997). et al.. Proteins: Struct., Funct., Genet..

[cit27] Hudson K. L. (2015). et al.. J. Am. Chem. Soc..

[cit28] Kiessling L. L., Diehl R. C. (2021). ACS Chem. Biol..

[cit29] Goodwin J. T., Lynn D. G. (1992). Angew. Chem., Int. Ed..

[cit30] Lane C. F. (1975). Synthesis.

[cit31] Corbett P. T., Sanders J. K. M., Otto S. (2008). Chem. – Eur. J..

[cit32] Mulla H. R., Agard N. J., Basu A. (2004). Bioorg. Med. Chem. Lett..

[cit33] Zhai W., Male L., Fossey J. S. (2017). Chem. Commun..

[cit34] Barclay T. (2012). et al.. Carbohydr. Res..

[cit35] Inoue K. (2011). et al.. Molecules.

