# Supplementary material for: Discovery of selective monosaccharide receptors via dynamic combinatorial chemistry
Source: Org Biomol Chem. 2024 Apr 12;22(19):3854–9. doi: 10.1039/d4ob00015c (PMC11095087; doi:10.1039/d4ob00015c)
Supplement: OB-022-D4OB00015C-s001 [file OB-022-D4OB00015C-s001.pdf]

Electronic Supplementary Material (ESI) for Organic & Biomolecular Chemistry.
This journal is © The Royal Society of Chemistry 2024

Electronic Supplementary Information (ESI) for:

Discovery of selective monosaccharide receptors via Dynamic Combinatorial Chemistry

Miguel Alena-Rodriguez,[a,b] *Marcos Fernandez-Villamarin,*[a] *Ignacio Alfonso*[b] *and Paula M Mendes*[*a]

[a] School of Chemical Engineering, University of Birmingham, Edgbaston, Birmingham, West Midlands, B15 2TT, UK. e-mail: p.m.mendes@bham.ac.uk

[b] Department of Biological Chemistry, Institute for Advanced Chemistry of Catalonia, IQAC-CSIC, Jordi Girona 18-26, 08034 Barcelona, Spain.

Table of Contents

General Methods S-3

General procedure for the preparation and analysis of DCC experiments S-4

General procedure for the synthesis of monosubstituted receptors 1D and 1F S-8

General procedure for the synthesis of disubstituted receptors 2DD and 2FF S-12

Isothermal titration calorimetry (ITC) binding studies S-16

Molecular modelling studies S-23

General Methods

General: Reagents and solvents were purchased from commercial suppliers (Aldrich or Merck) and were used without further purification.

Reversed-Phase High-Performance Liquid Chromatography (RP-HPLC) HPLC purification was performed with Agilent Technologies 1260 Infinity systems. The column used was an AerisTM WIDEPORE (C18, 150 x 4.6 mm) with 3.6 µm particle size and 200 Å pore size and the monitoring wavelengths were set at 220 and 254 nm.

Liquid Chromatography-Mass Spectrometry (LC-MS) analyses for the screening of the DCLs were performed with a Waters SQD 2 (MS) instrument connected to a Waters Alliance e2695 (LC) and UV detector in the range 210 to 500 nm, working with electrospray ionization (positive mode). Kinetex Phenyl-Hexyl 5 µm 100A (250x4.6 mm) column was employed. LC-MS data was analysed with MassLynx software.

Nuclear Magnetic Resonance (NMR) Proton NMR spectra were recorded at 400 MHz on a Bruker AVIII400 NMR spectrometer. Carbon NMR spectra are proton decoupled and were recorded at 100 MHz on a Bruker AVIII400 NMR spectrometer at room temperature. The chemical shifts are reported in ppm relative to trimethylsilane (TMS) and coupling constants (J) are reported in Hertz (Hz). MestReNova software was used for the analysis of the spectrums.

High Resolution Mass Spectroscopy (HRMS) Mass spectra were recorded with a Waters Xevo G2-XS spectrometer working with electrospray ionization (positive mode).

Isothermal Titration Calorimetry (ITC) ITC binding studies were conducted on a TA nano-LV instrument running in aqueous mode. The corresponding isotherms were analysed using TA NanoAnalyze software using the independent model and n value fixed to 1.

General procedure for the preparation and analysis of DCC experiments

2 (1 mM) was mixed with A, B, W, F, and D (4 mM each) in 100 mM carbonate buffer pH 10 90%-10% MeCN. Then, the reaction mixture was split into five 1 mL Eppendorf tubes and to each one of them it was added 10 μL of a water solution of one sugar (glucose, mannose, galactose, or fructose, 2 mM in reaction mixture); as well as the same volume of distilled water to the fifth tube. The mixtures were left stirring O.N. Then, $NaCNBH_3$ (20 mM in the reaction mixture) was added to the Eppendorf tubes and they were stirred for 30 min before LC-MS analysis.

Then, the samples were analyzed by LC-MS. The mobile phase consisted of A: Water + 0.1% formic acid and B: methanol + 0.1% formic acid. The running gradient was the following:

Time	A%	B%	Flow (mL/min)
0.00	95.0	5.0	1.000
1.00	95.0	5.0	1.000
11.00	5.0	95.0	1.000
12.00	5.0	95.0	1.000
12.10	95.0	5.0	1.000
15.00	95.0	5.0	1.000

In total, there were five different DCLs to be screened by LC-MS: the four templated ones (with glucose, galactose, mannose, and fructose), as well as the non-templated DCL (blank). Each DCL was screened three times.

The m/z of the $(M+H)^+$ ion for each and every one of the library members was searched in the extracted ion chromatogram (EIC) for all the LC-MS runs.

In the case of monofunctionalised library members, there was the possibility of $NaCHBH_3$ reducing either the imine (2X, figure 2), the unreacted aldehyde, or both groups (2X-OH, Figure 2). LC-MS confirmed the absence of 2X-OH. Both mono-reduced products would be indistinguishable by LC-MS by looking at their m/z as they would have the same mass. However, the product of reduction of starting aldehyde 2 was not detected by LC-MS, suggesting that in the conditions employed, $NaCNBH_3$ did not reduce the aromatic aldehydes. Therefore, we can conclude that the mono-reduced library members are indeed the secondary amines 2X.

The intensity of the peaks (measured as the number of ions detected on the EIC) for each library member in a templated DCL was divided by the intensity of the same peak in the blank

experiment. The greater the value obtained as a result of such operation, the larger the amplification caused by such template to that library member.

As examples, the LC-MS UV and TIC chromatograms, as well as the EIC for the library member 2DD in the blank run as well as in the four templated runs are included here (Figure S1-S4). The same procedure for the EIC of 2F with fructose is attached too (Figure S5).

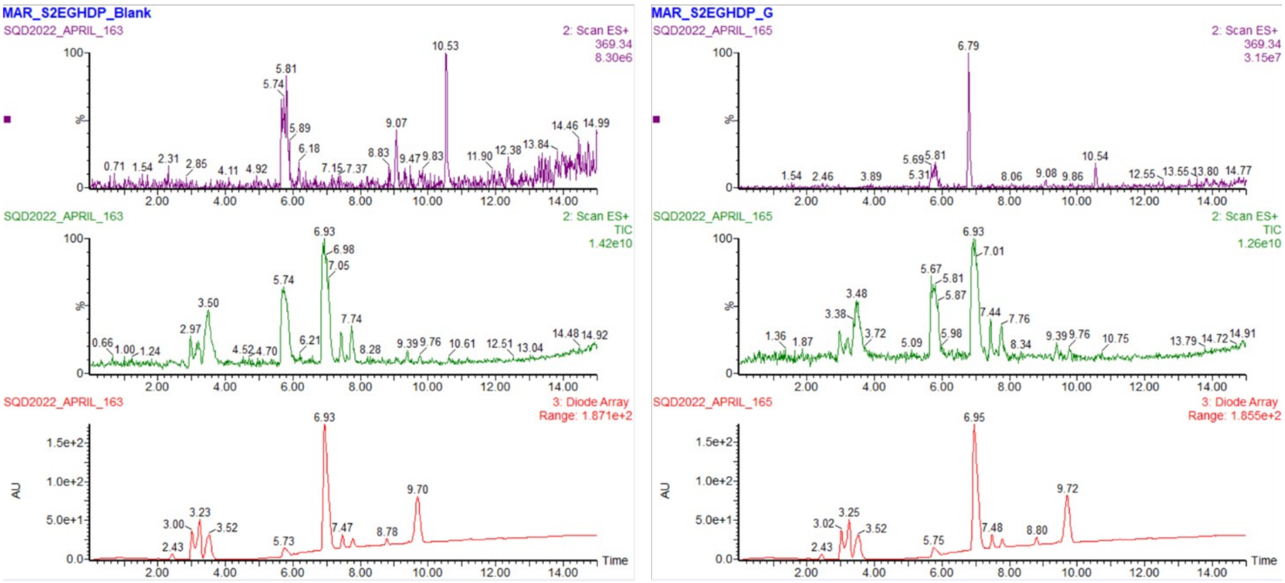

Figure S1: UV (bottom, red). TIC (middle, green) and EIC (top, purple) chromatograms for the LC-MS runs of the untemplated DCC (left) and the DCC templated with D-glucose (right). The m/z shown in the EIC chromatogram is the $(M+H)^+$ of molecule 2DD (369.34) and the intensity of the peaks are 8.30e6 and 3.15e7 for non-templated and templated runs, respectively.

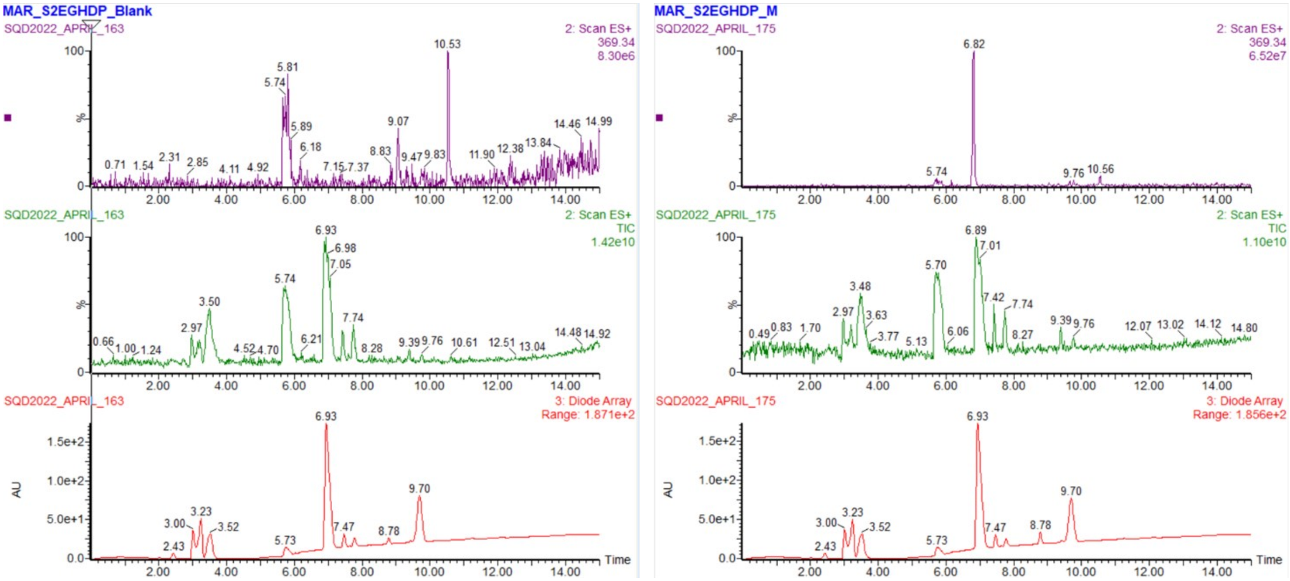

Figure S2: UV (bottom, red). TIC (middle, green) and EIC (top, purple) chromatograms for the LC-MS runs of the untemplated DCC (left) and the DCC templated with D-mannose (right). The m/z shown in the EIC chromatogram is the $(M+H)^+$ of molecule 2DD (369.34) and the intensity of the peaks are 8.30e6 and 6.52e7 for non-templated and templated runs, respectively.

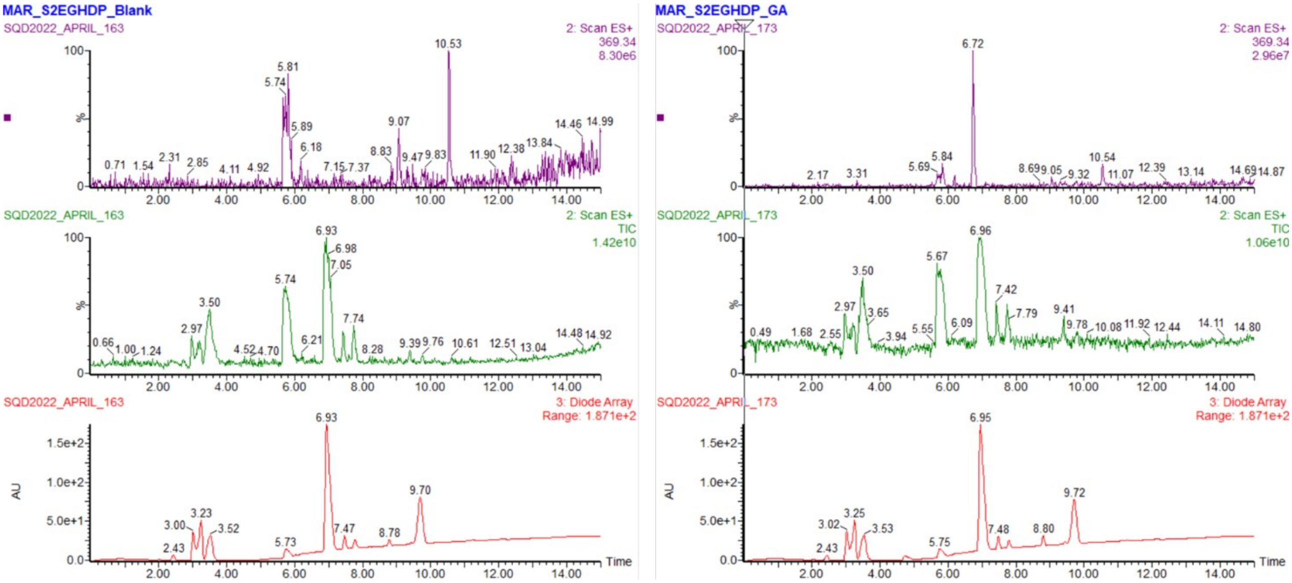

Figure S3: UV (bottom, red). TIC (middle, green) and EIC (top, purple) chromatograms for the LC-MS runs of the untemplated DCC (left) and the DCC templated with D-galactose (right). The m/z shown in the EIC chromatogram is the $(M+H)^+$ of molecule 2DD (369.34) and the intensity of the peaks are 8.30e6 and 2.96e7 for non-templated and templated runs, respectively.

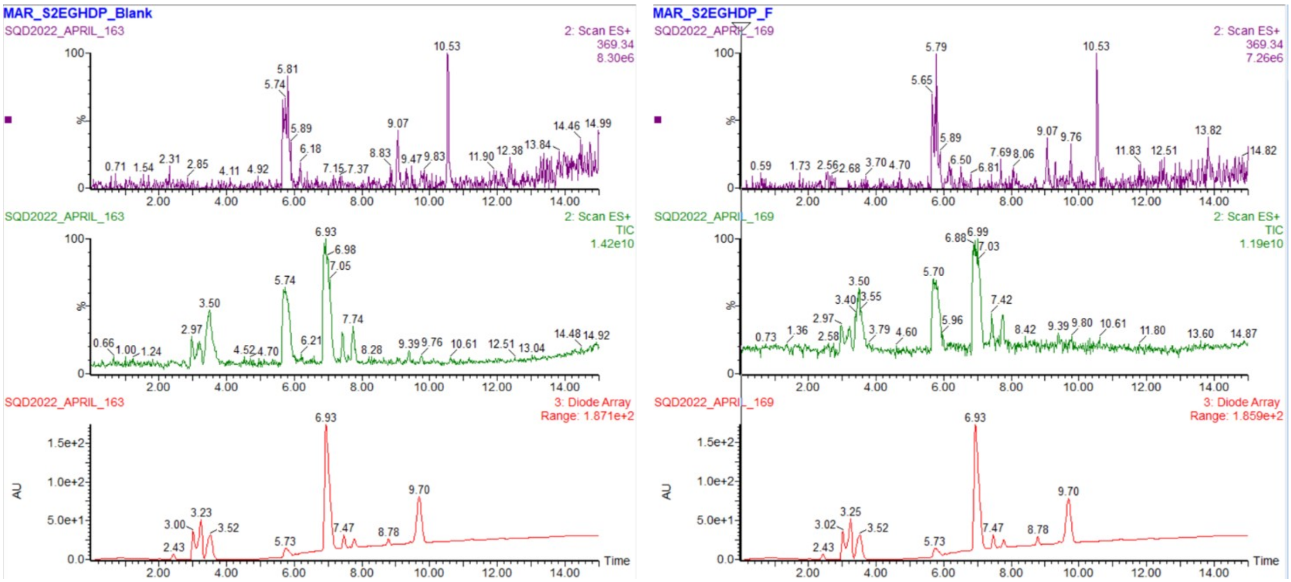

Figure S4: UV (bottom, red). TIC (middle, green) and EIC (top, purple) chromatograms for the LC-MS runs of the untemplated DCC (left) and the DCC templated with D-fructose (right). The m/z shown in the EIC chromatogram is the $(M+H)^+$ of molecule 2DD (369.34) and the intensity of the peaks are 8.30e6 and 7.25e6 for non-templated and templated runs, respectively.

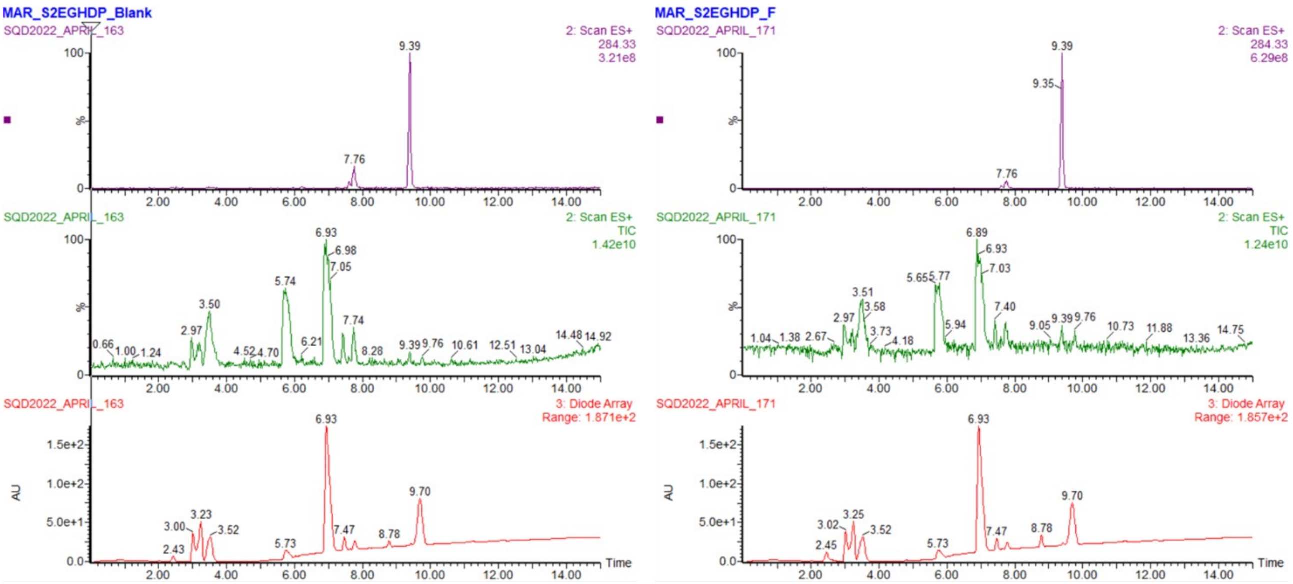

Figure S5: UV (bottom, red). TIC (middle, green) and EIC (top, purple) chromatograms for the LC-MS runs of the untemplated DCC (left) and the DCC templated with D-fructose (right). The m/z shown in the EIC chromatogram is the $(M+H)^+$ of molecule 2F (284.33) and the intensity of the peaks are 3.21e8 and 6.29e8 for non-templated and templated runs, respectively.

General procedure for the synthesis of monosubstituted receptors 1D and 1F

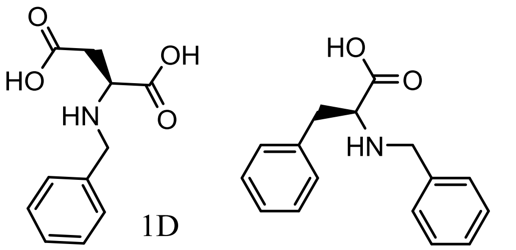

1D 1F

L-Aspartic acid (**D**, 652.0 mg, 4.9 mmol) or L-phenylalanine (**F**, 821.7 mg, 4.9 mmol) was slowly dissolved in a solution of NaOH (0.4 g) in water (5 mL) and methanol (10 mL). Then, benzaldehyde (**1**, 508 uL, 5.0 mmol) was slowly added to the solution. The reaction mixture was stirred for 1h. $NaBH_4$ (226.0 mg, 6 mmol) was dissolved in methanol (1 mL) and slowly added to the reaction mixture. Reaction left stirring for 1h before acidifying it to pH 5-6 with acetic acid. The solvent was evaporated under reduced pressure. The resulted oils were purified by HPLC to afford **1D** (907.2 mg, 83%) and **1F** (950.1 mg, 76%) as white solids. HPLC purification method was performed on C18 column and was optimised as follows: A 95%-5% B isocratic for 25 minutes, with a retention time for **1D** of 9.1 minutes and for **1F** of 20.0 minutes. Being A: water + 0.1% TFA and B: acetonitrile + 0.1% TFA.

1D: ^1H NMR (400 MHz, D_2O, 298 K) δ 7.48 (s, 5H), 4.38 (d, *J = 13.1 Hz*, 1H), 4.32 (d, *J = 13.1 Hz*, 1H), 4.18 (t, *J = 5.6 Hz*, 1H), 3.08 (dd, *J = 17.6, 3.9 Hz*, 2H). ^{13}C NMR (100 MHz, D_2O, 298 K) δ 170.79, 130.02, 129.28, 55.84, 50.63, 33.49. HRMS (ESI+): calculated for $C_{11}H_{14}NO_4$ $[M+H]^+$: 224.0923, found: 224.0929.

1F: ^1H NMR (400 MHz, D_2O, 298 K) δ 7.45-7.20 (m, 10H), 3.61 (d, *J = 13.1 Hz*, 1H), 3.40 (d, J = *13.1 Hz*, 1H), 2.92 (m, 2H), 2.63 (m, 1H). ^{13}C NMR (100 MHz, D_2O, 298 K) δ 181.15, 138.84, 138.07, 129.28, 128.65, 128.53, 127.36, 126.60, 64.47, 51.01, 39.03. HRMS (ESI+): calculated for $C_{16}H_{18}NO_2$ $[M+H]^+$: 256.1338, found: 256.1342.

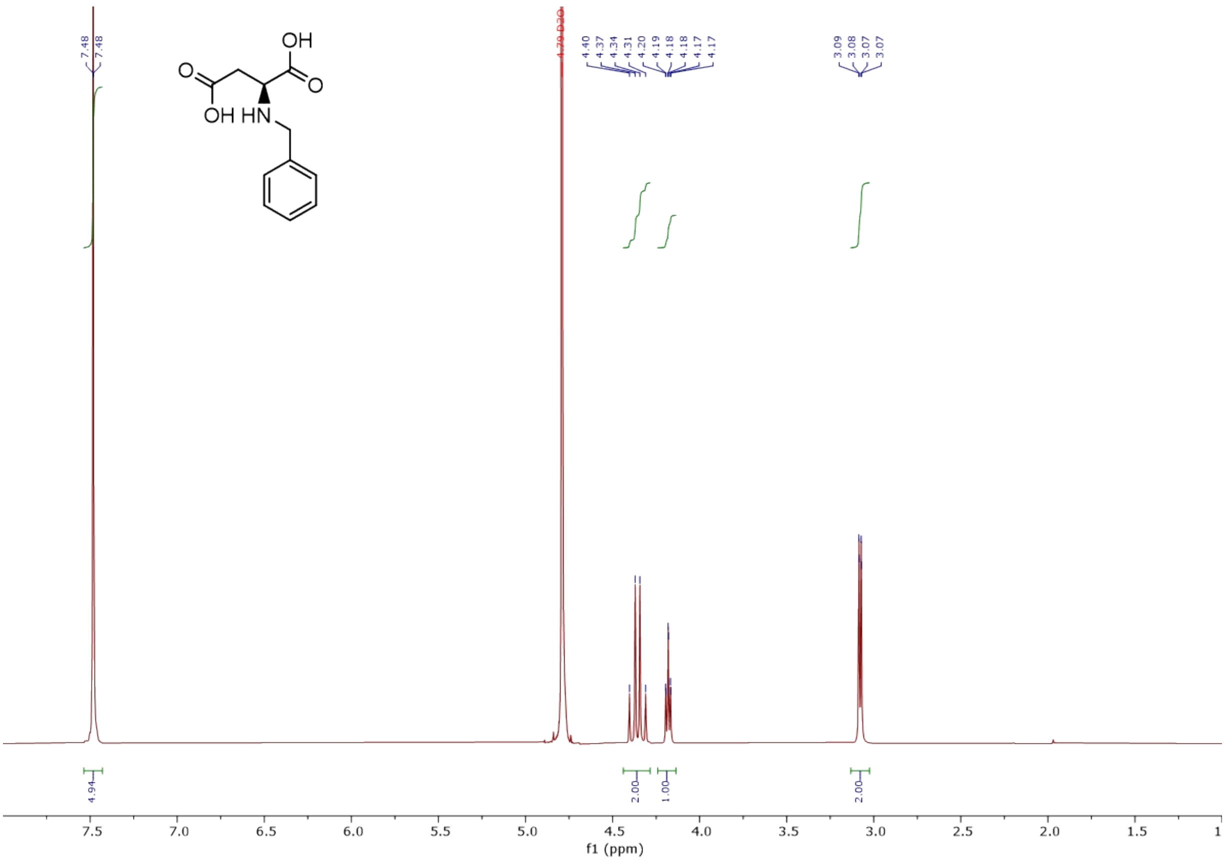

Figure S6: ^{1}H NMR spectrum of **1D** (400 MHz, D_2O, 298 K).

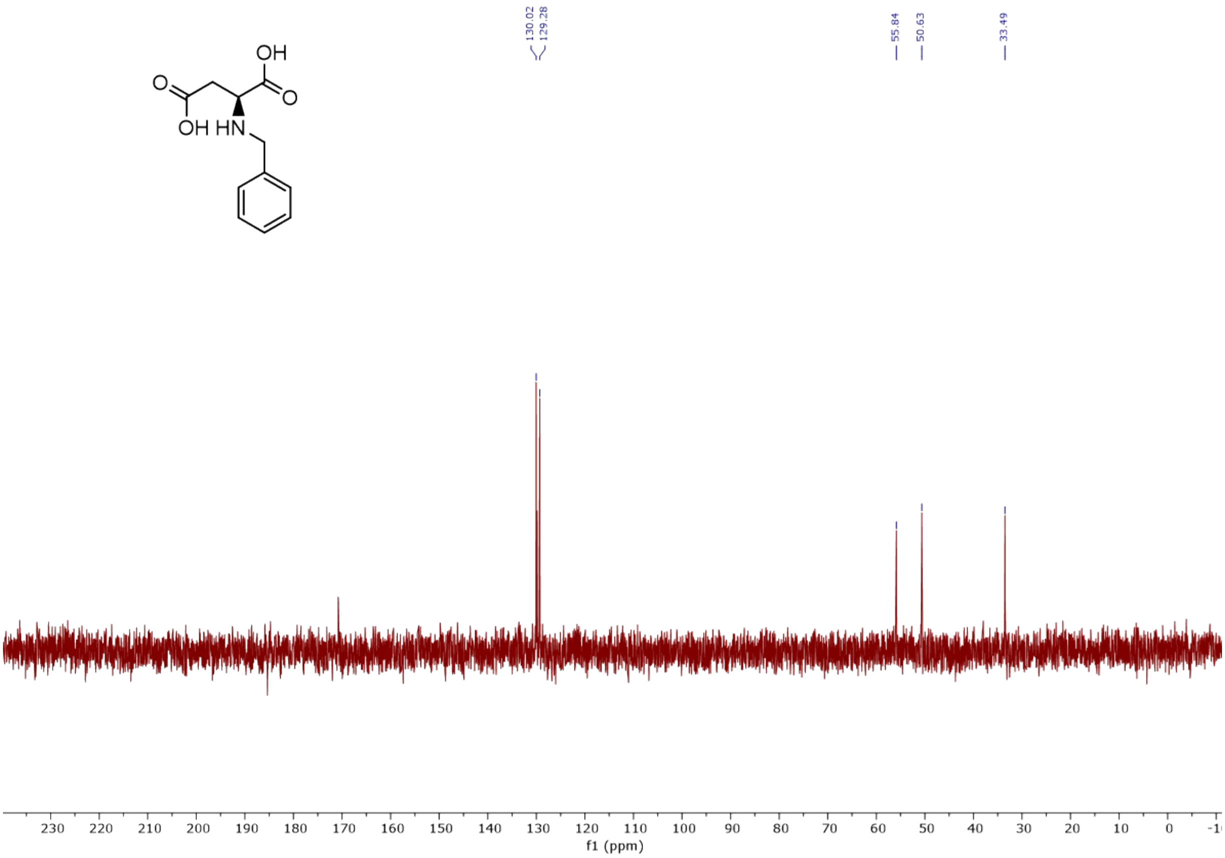

Figure S7: ^{13}C NMR spectrum of **1D** (100 MHz, D_2O, 298 K).

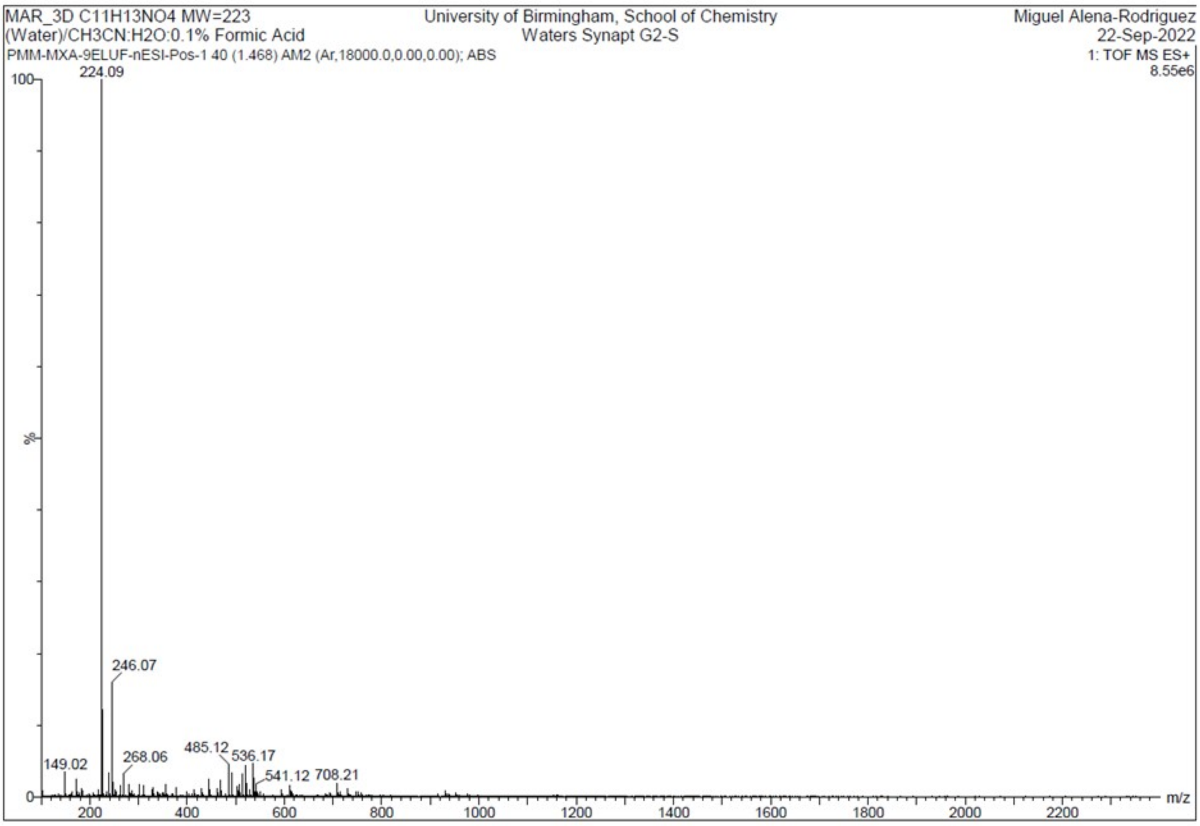

Figure S8: HRMS (ESI+) spectrum of **1D.**

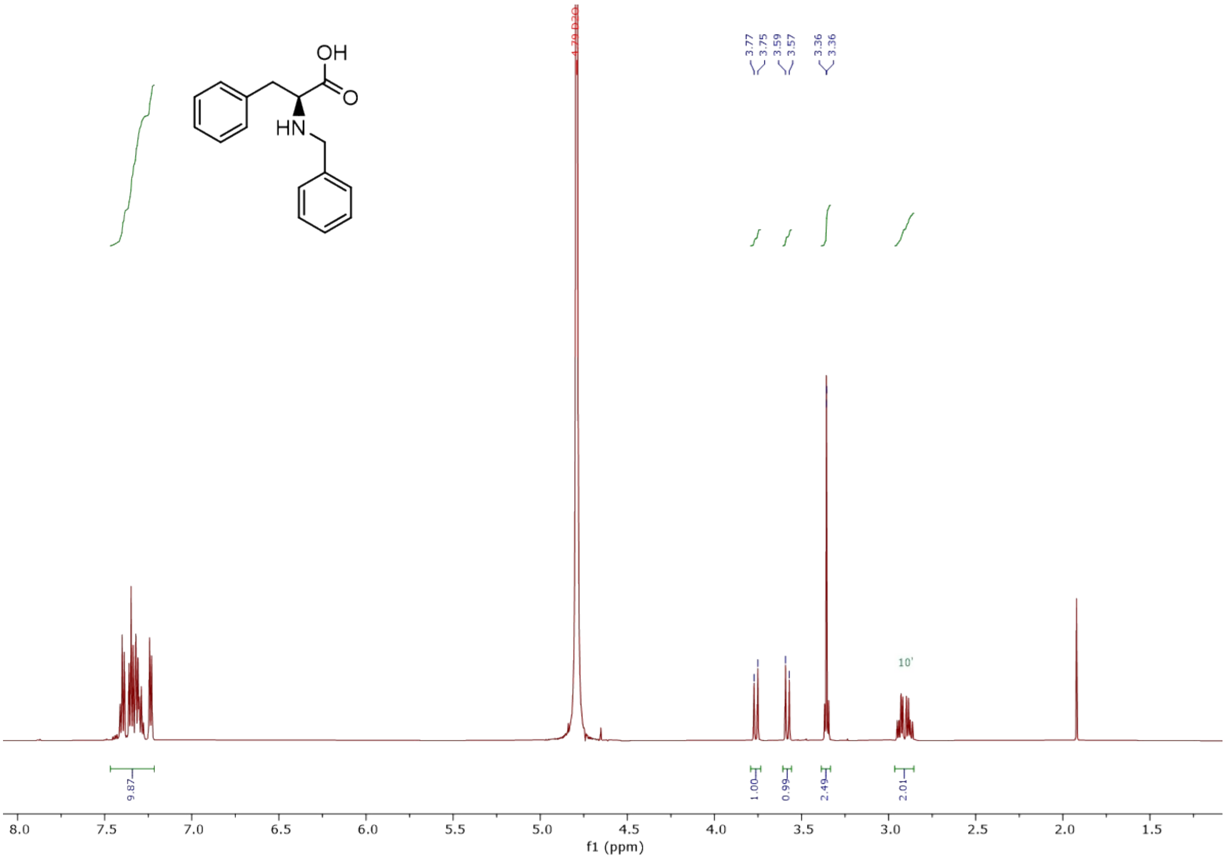

Figure S9: ^{1}H NMR spectrum of **1F** (400 MHz, D_2O, 298 K).

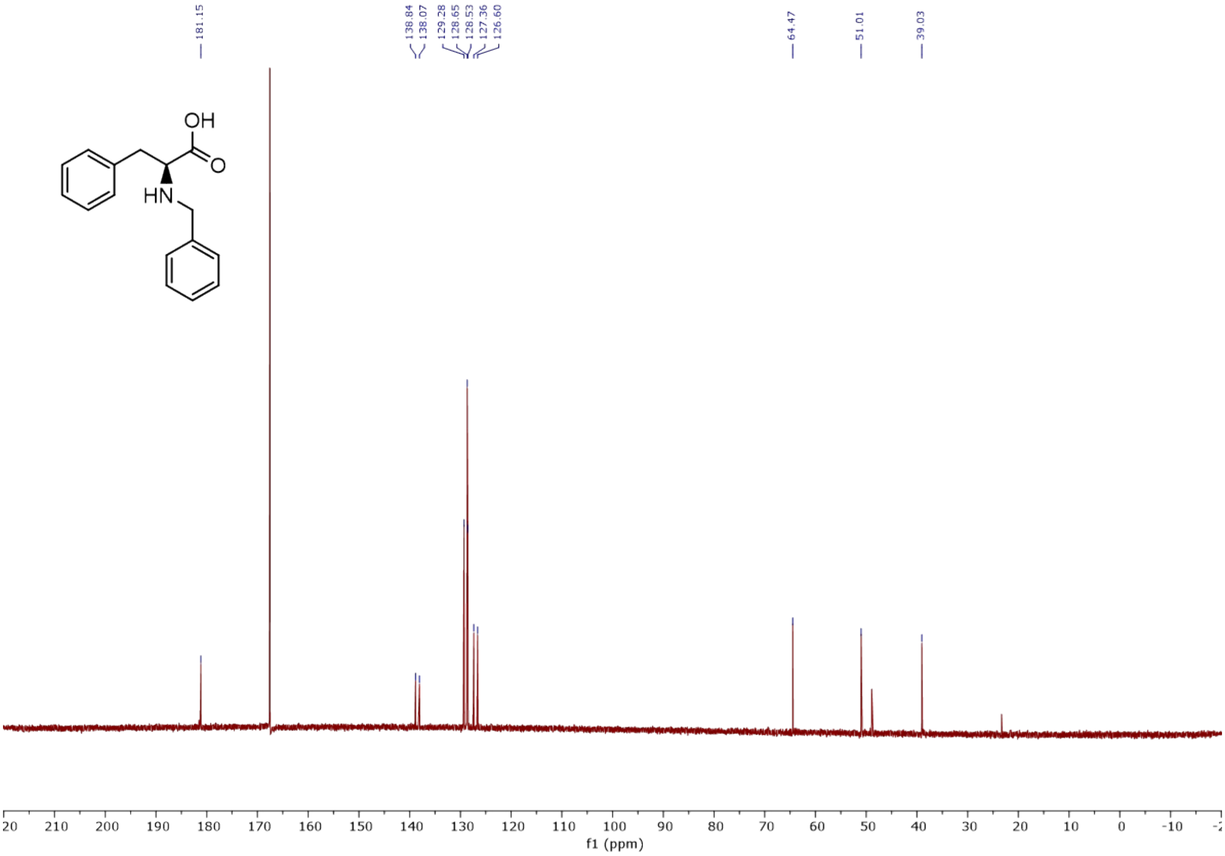

Figure S10: ^{13}C NMR spectrum of **1F** (100 MHz, D_2O, 298 K).

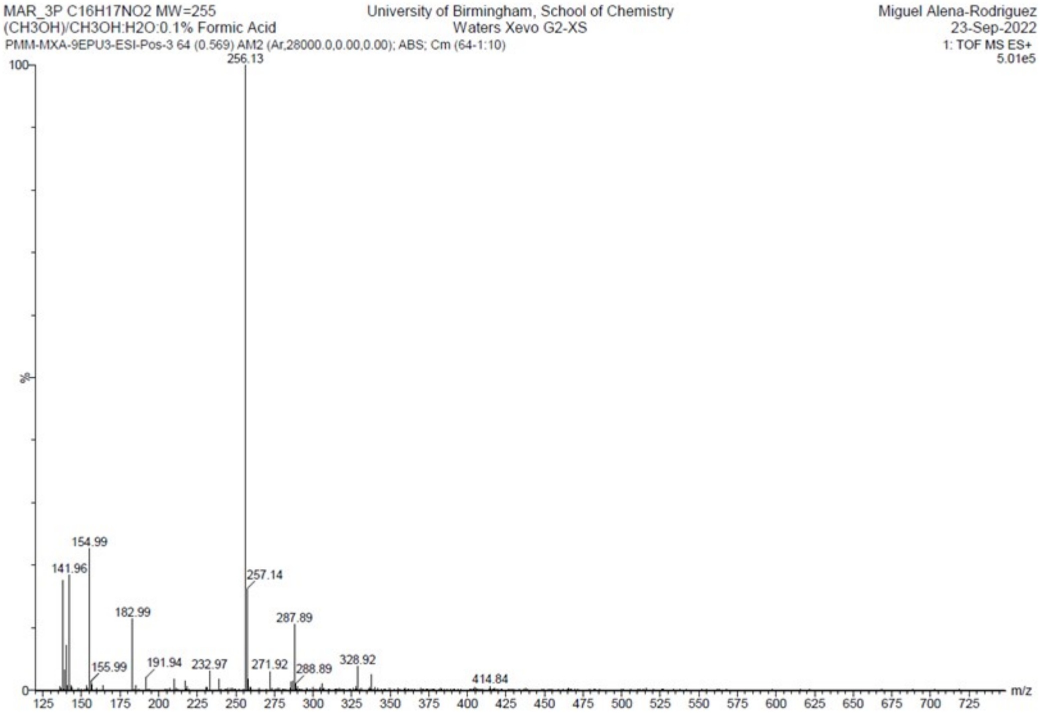

Figure S11: HRMS (ESI+) spectrum of **1F**.

General procedure for the synthesis of disubstituted receptors 2DD and 2FF

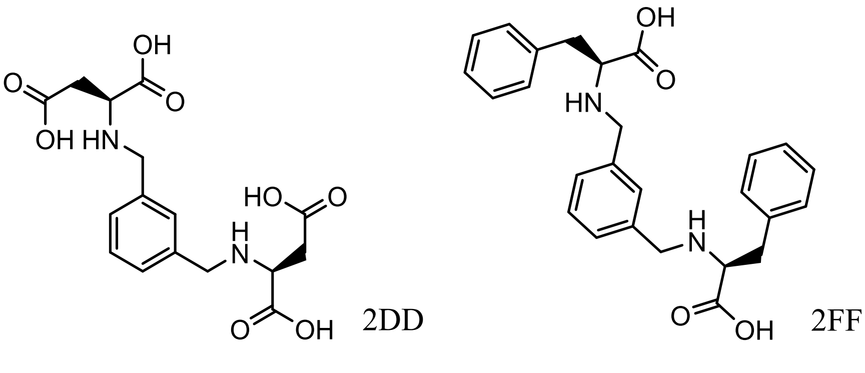

2DD

2FF

L-Aspartic acid (**D**, 665.3 mg, 5.0 mmol) or L-phenylalanine (**F**, 825.9 mg, 5.0 mmol) was dissolved in a solution of NaOH (0.4 g) in water (5 mL) and methanol (10 mL). Then, isophthalaldehyde (**2**, 335.0 mg, 2.5 mmol) was dissolved in methanol (10 mL) and slowly added to the amino acid solution. The reaction mixture was stirred for 6h. $NaBH_4$ (226.0 mg, 6 mmol) was dissolved in methanol (1 mL) and slowly added to the reaction mixture. Reaction left stirring for 1h before acidifying it to pH 5-6 with acetic acid. The solvent was evaporated under reduced pressure. The resulted oil was purified by HPLC to afford **2DD** (552.1 mg, 60%) and **2FF** (713.1 mg, 66%) as white solids. HPLC purification method was optimised as follows: A 95%-5% B isocratic for 15 minutes, with a retention time for **2DD** of 5.1 minutes and for **2PP** of 10.1 minutes. Being A: water + 0.1% TFA and B: acetonitrile + 0.1% TFA.

2DD: ^{1}H NMR (400 MHz, D_2O, 298 K) δ 7.36 (t, *J= 7.64 Hz*, 1H), 7.28 (s, 1H), 7.27(d, *J = 7.64 Hz*, 2H), 3.72 (d, *J = 13.1 Hz*, 2H), 3.61 (d, *J = 13.1 Hz*, 2H), 3.43 (dd, *J = 8.13, 5.63 Hz*, 2H), 2.50 (dd, *J = 15.13, 5.64 Hz*, 2H), 2.34 (dd, *J = 15.15, 8.14 Hz*, 2H). HRMS (ESI+): calculated for $C_{16}H_{19}N_2O_8$ $[M+H]^+$: 367.1141, found: 367.1139.

2FF: ^{1}H NMR (400 MHz, D_2O, 298 K) δ 7.35-7.22 (m, 14H), 3.73 (d, *J = 13.1 Hz*, 2H), 3.55 (d, *J = 13.1 Hz*, 2H), 3.35 (t, *J = 6.98 Hz*, 2H), 2.91 (m, 4H). ^{13}C NMR (100 MHz, D_2O, 298 K) δ 181.17, 139.09, 138.09, 129.27, 128.71, 128.53, 128.44, 127.42, 126.60, 117.78, 114.89, 64.48, 50.87, 39.04. HRMS (ESI+): calculated for $C_{26}H_{27}N_2O_4$ $[M+H]^+$: 431.1971, found: 431.1983.

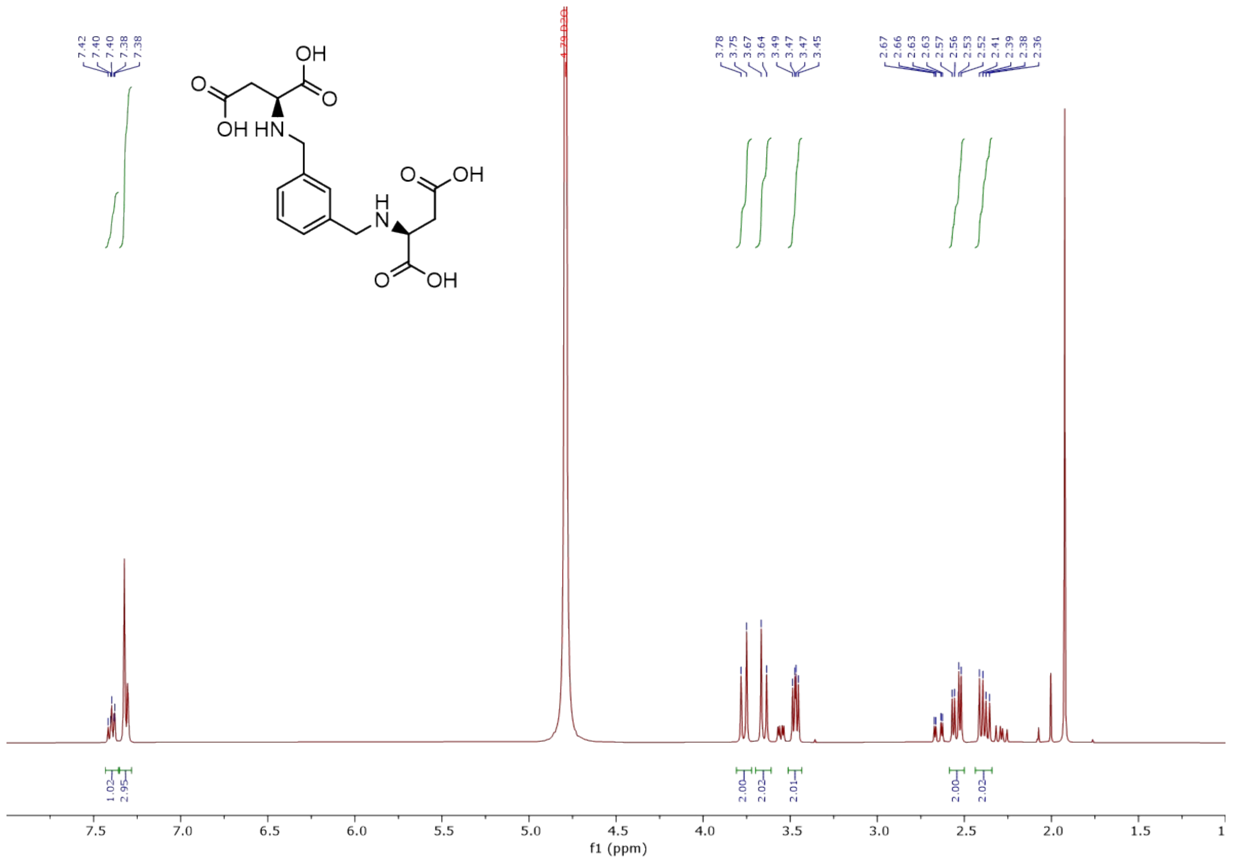

Figure S12: ^{1}H NMR spectrum of **2DD** (400 MHz, D_2O, 298 K).

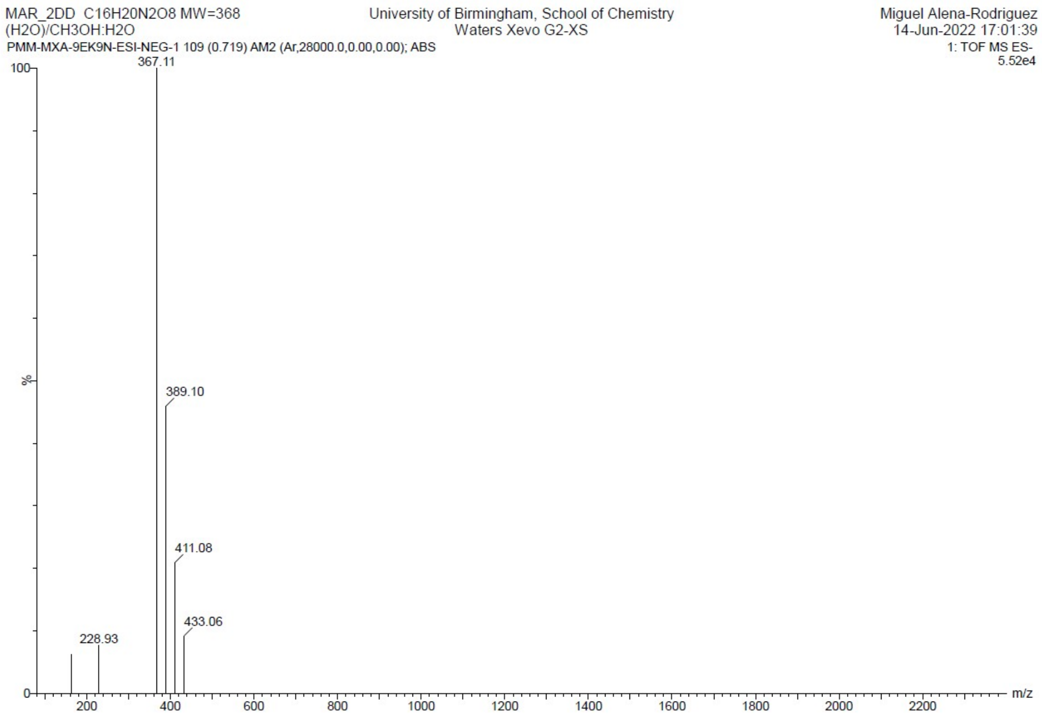

Figure S13: HRMS (ESI+) spectrum of **2DD.**

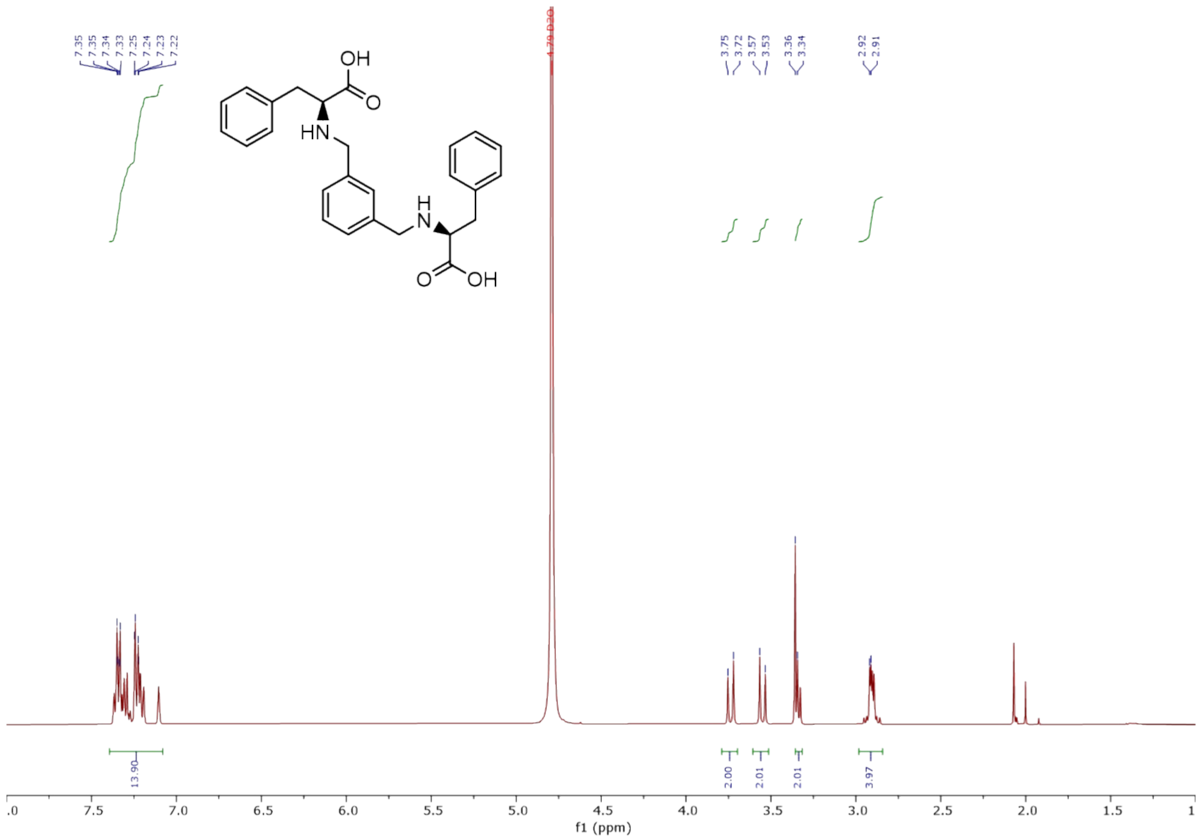

Figure S14: ^{1}H NMR spectrum of **2FF** (400 MHz, D_2O, 298 K).

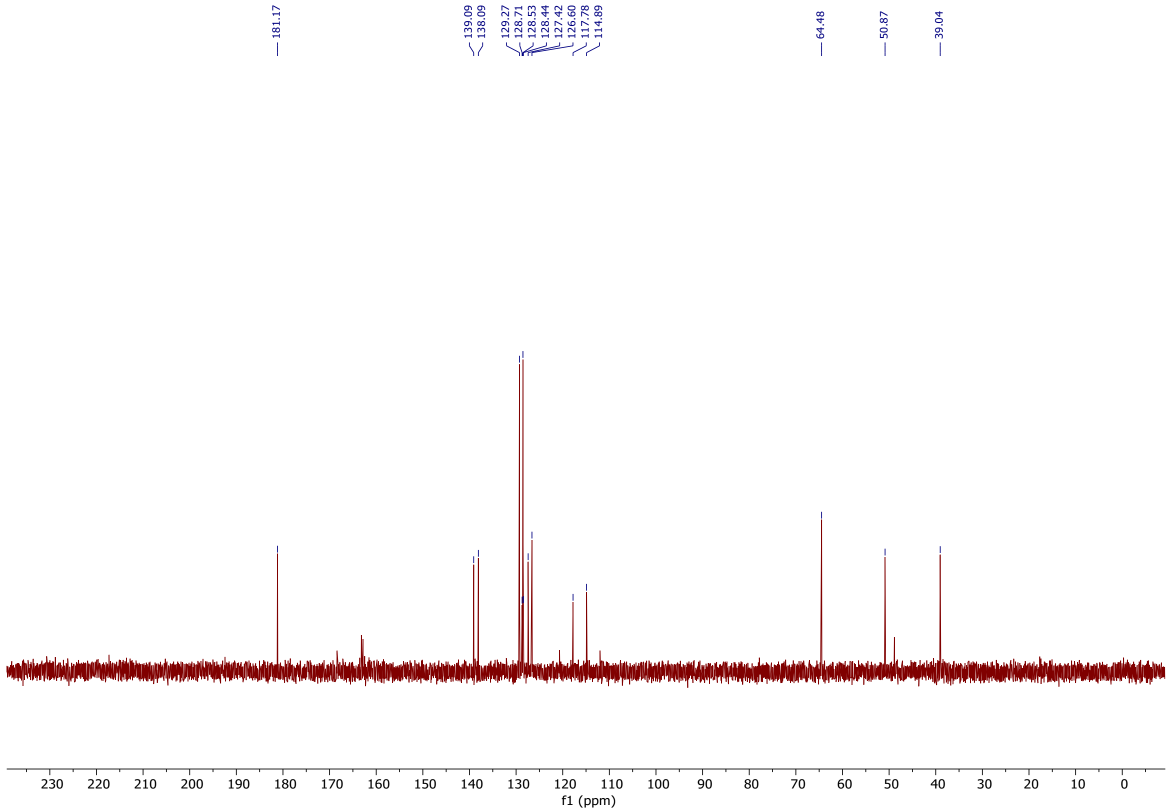

Figure S15: ^{13}C NMR spectrum of **2FF** (100 MHz, D_2O, 298 K).

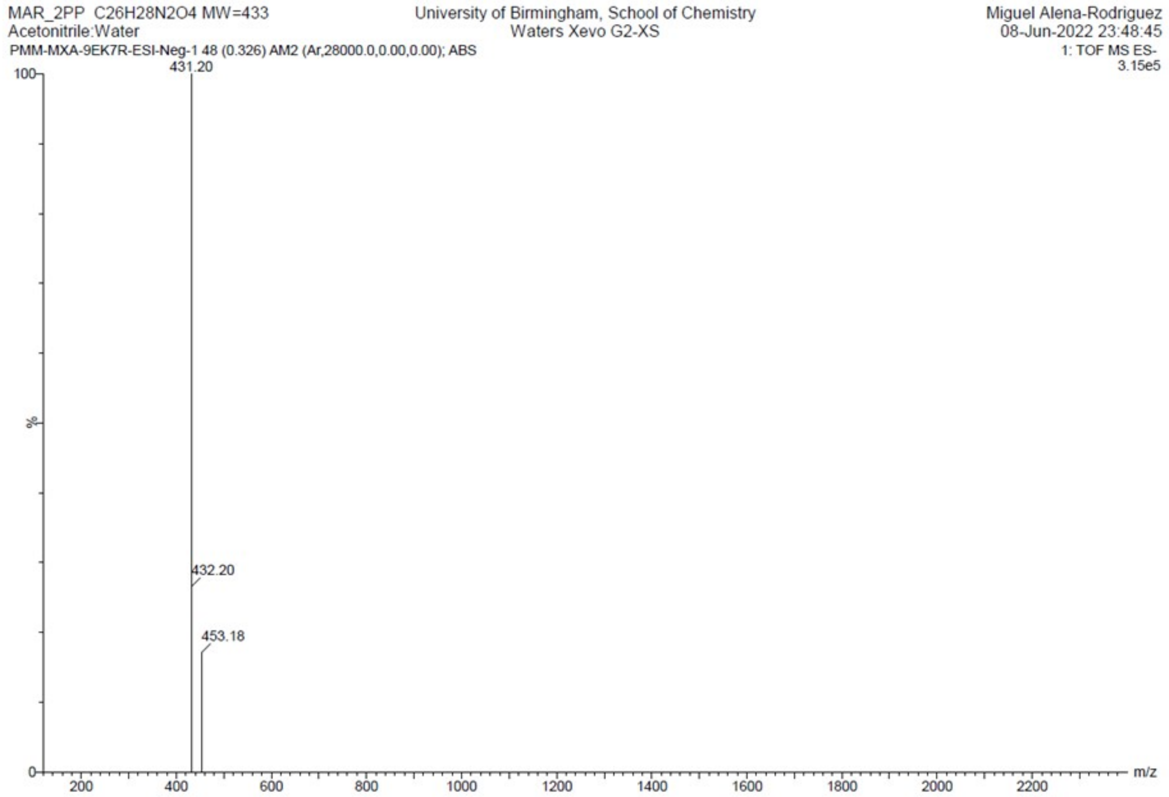

Figure S16: HRMS (ESI+) spectrum of **2FF.**

Isothermal titration calorimetry (ITC) binding studies

ITC experiments were performed with 2 mM receptor solution (D, F, 1D, 1F, 2DD, 2FF) and 80 mM solution of ligand (D-glucose, D-mannose, D-galactose, D-fructose). 100 mM carbonate buffer (pH 10) was employed as solvent. The buffer was degassed prior to solution preparation. The solutions were filtered and degassed prior to use. ITC experiments were performed with the parameters reported in Table S1 by titrating the ligand into the receptor solution. Each experiment consists of 3 titrations and the heat of dilution was measured and subtracted for each experiment.

Table S1. ITC parameters for the binding studies with TA nano-LV instrument.

Ligand concentration	80 mM
Receptor concentration	2 mM
Number of injections	40
Injection volume	1.5 µL
Duration of each injection	4.0 s
Initial cell volume	185 µL
Cell temperature	20 °C
Initial delay	60 s
Stirring speed	150 RPM
Spacing	280 - 480 s

2DD - Glucose. ITC replicate 1

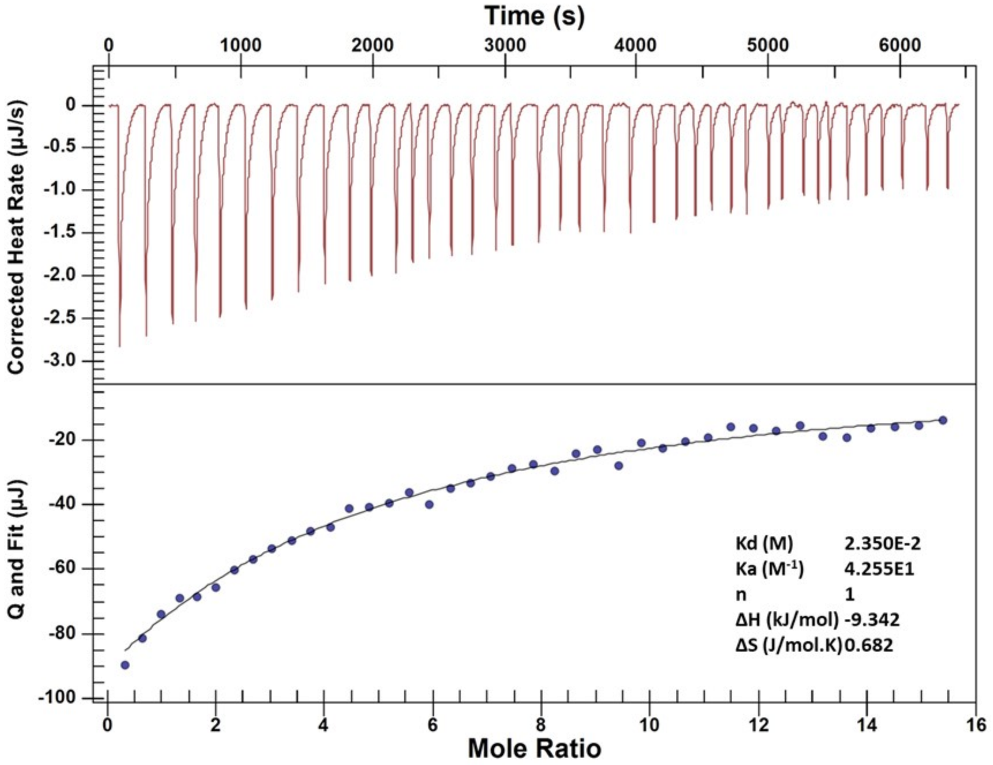

Figure S17: ITC titration of **2DD** and glucose.

1F - Galactose. ITC replicate 1

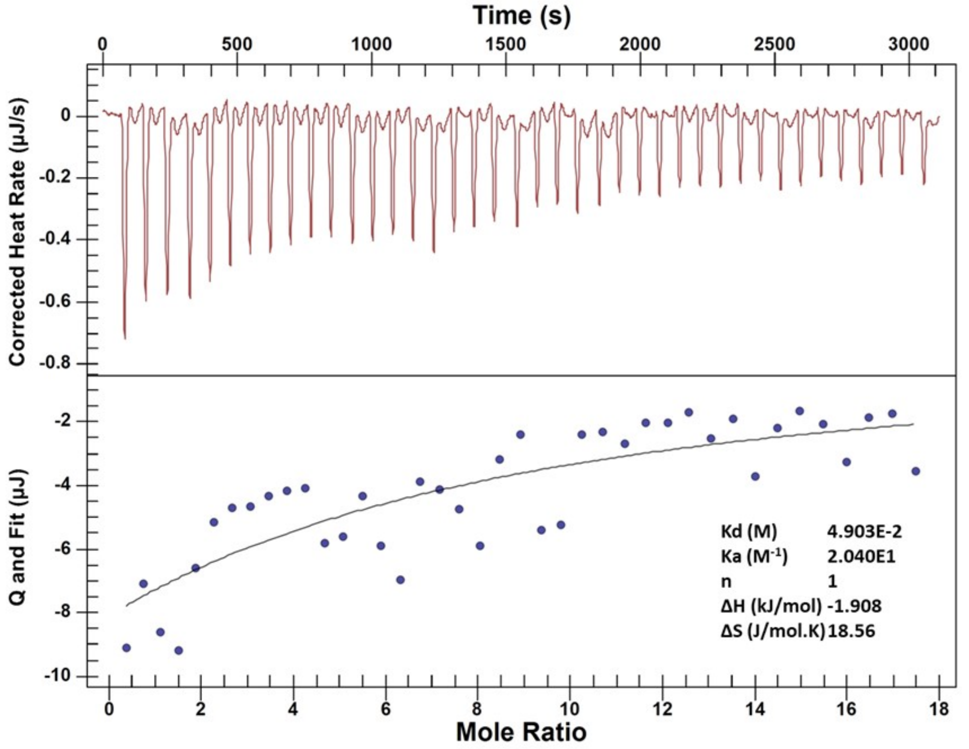

Figure S18: ITC titration of **1F** and galactose.

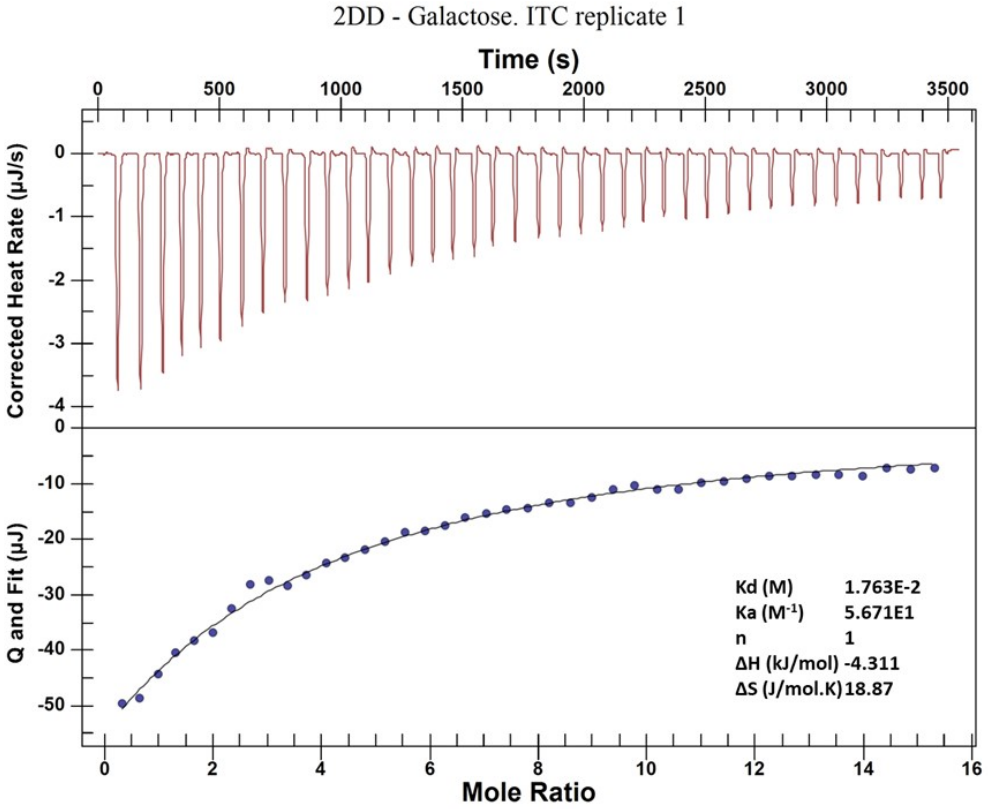

Figure S19: ITC titration of **2DD** and galactose.

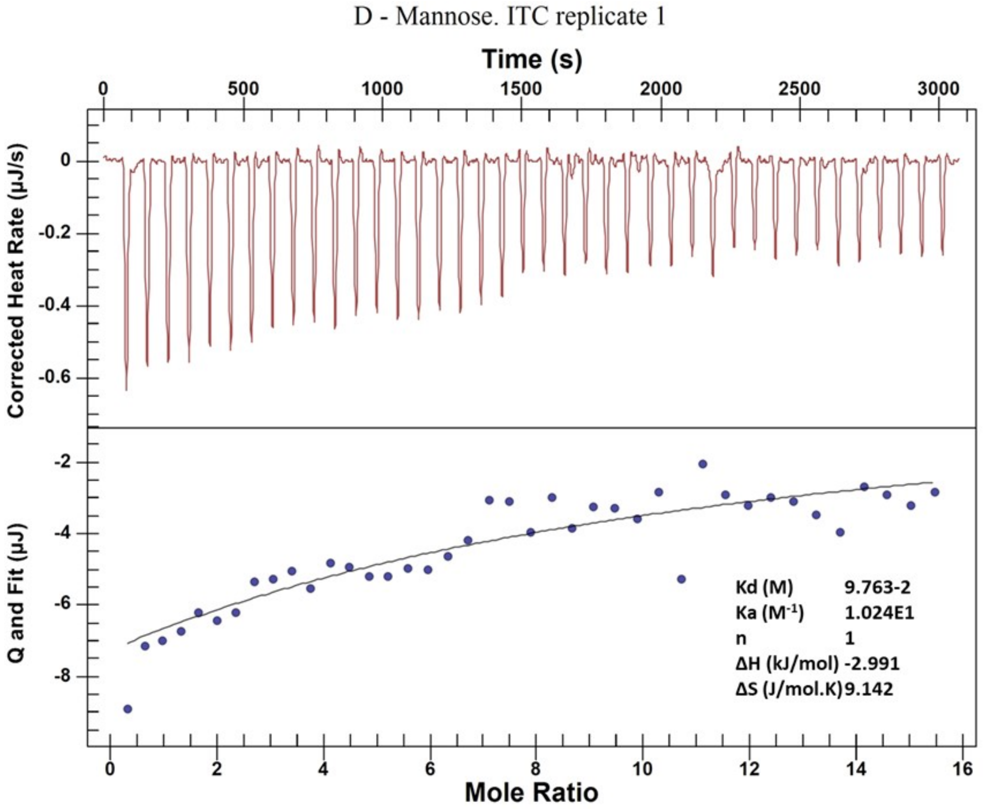

Figure S20: ITC titration of **D** and mannose.

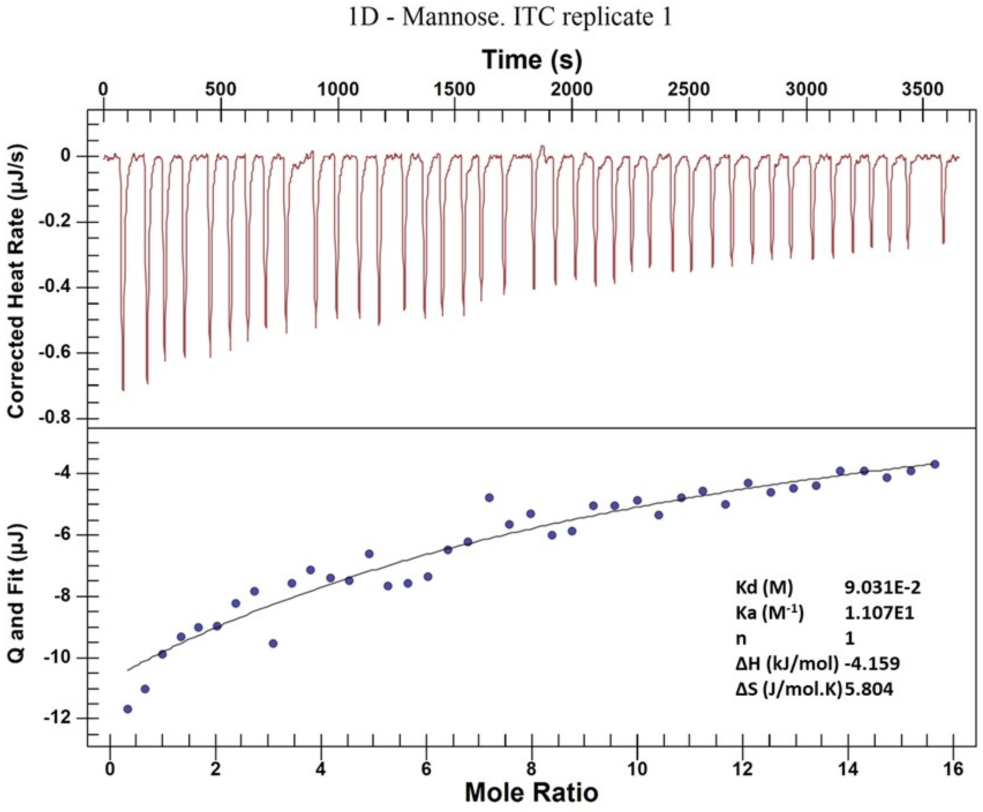

Figure S21: ITC titration of **1D** and mannose.

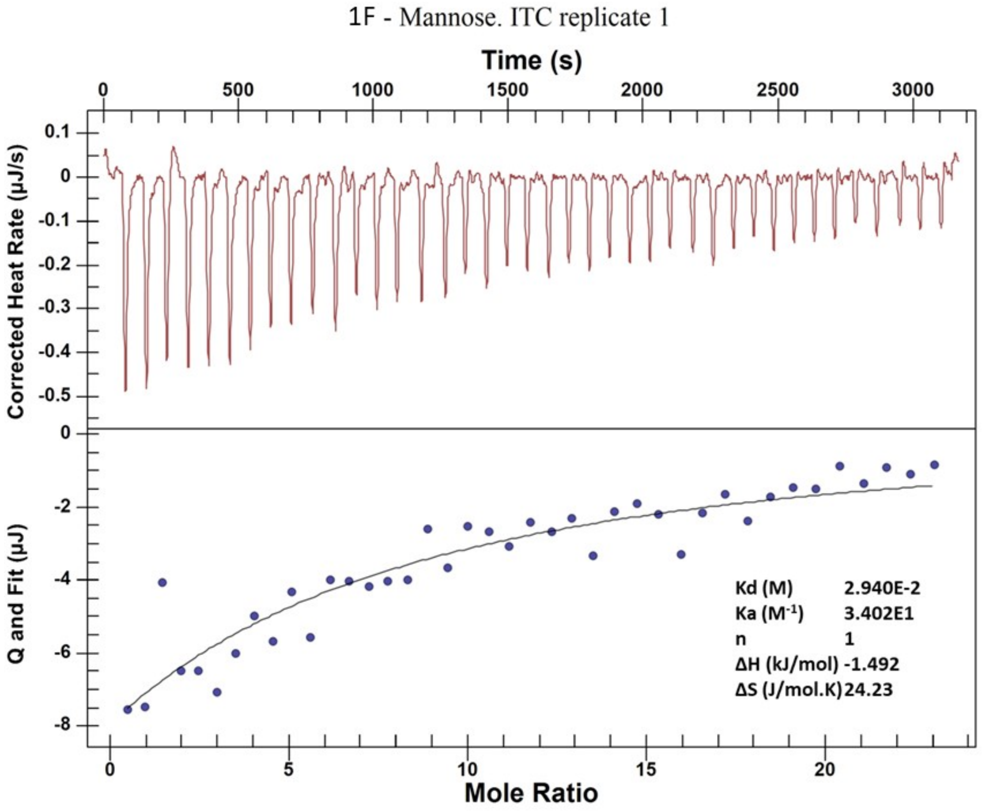

Figure S22: ITC titration of **1F** and mannose.

2DD - Mannose. ITC replicate 1

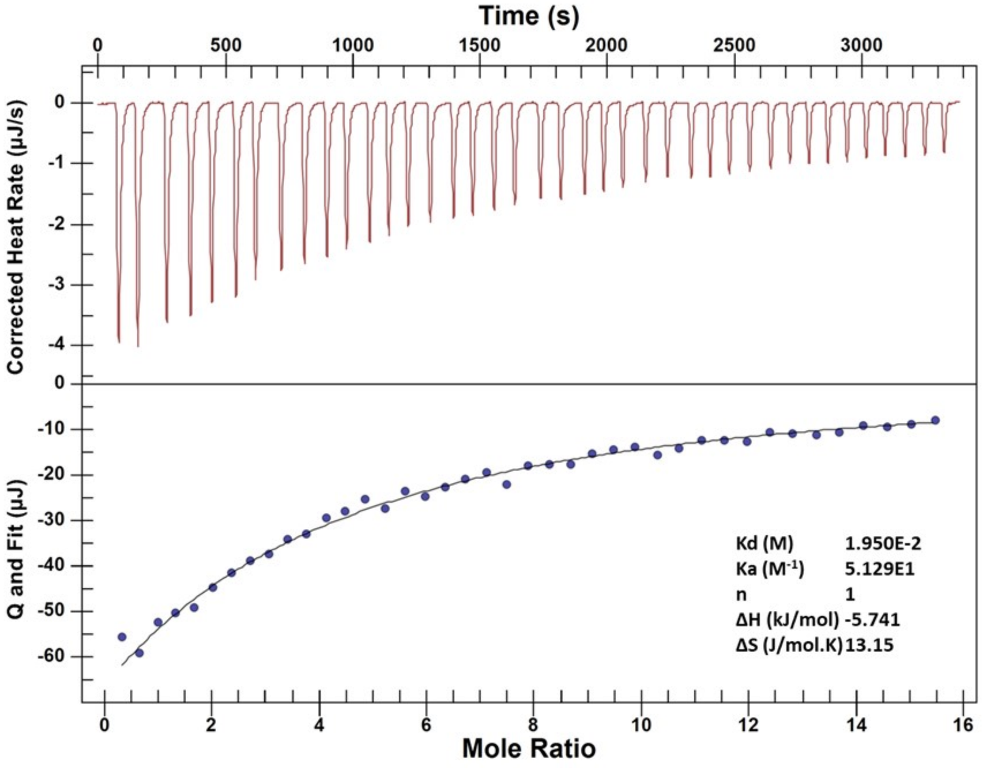

Figure S23: ITC titration of **2DD** and mannose.

F - Fructose. ITC replicate 1

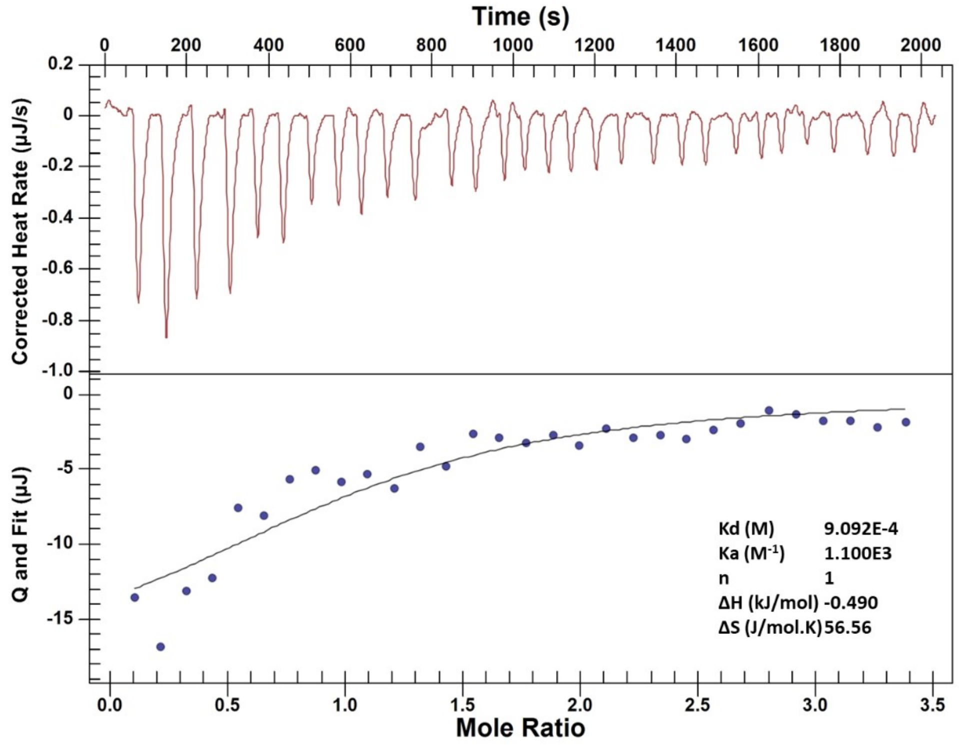

Figure S24: ITC titration of **F** and fructose.

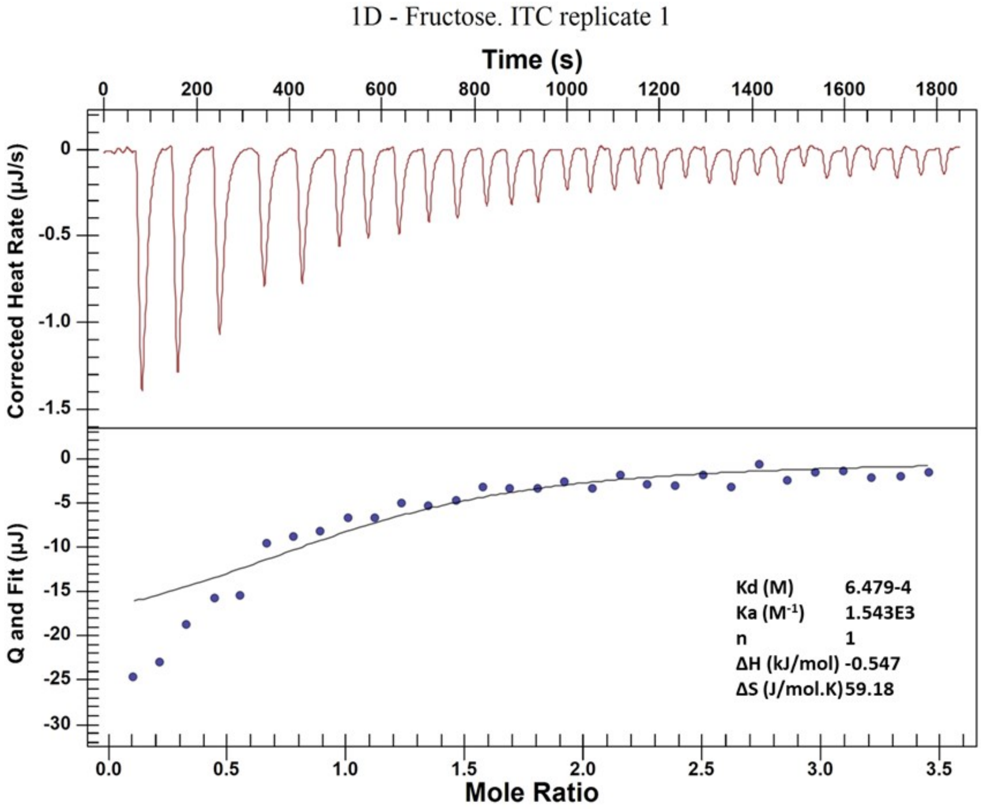

Figure S25: ITC titration of **1D** and fructose.

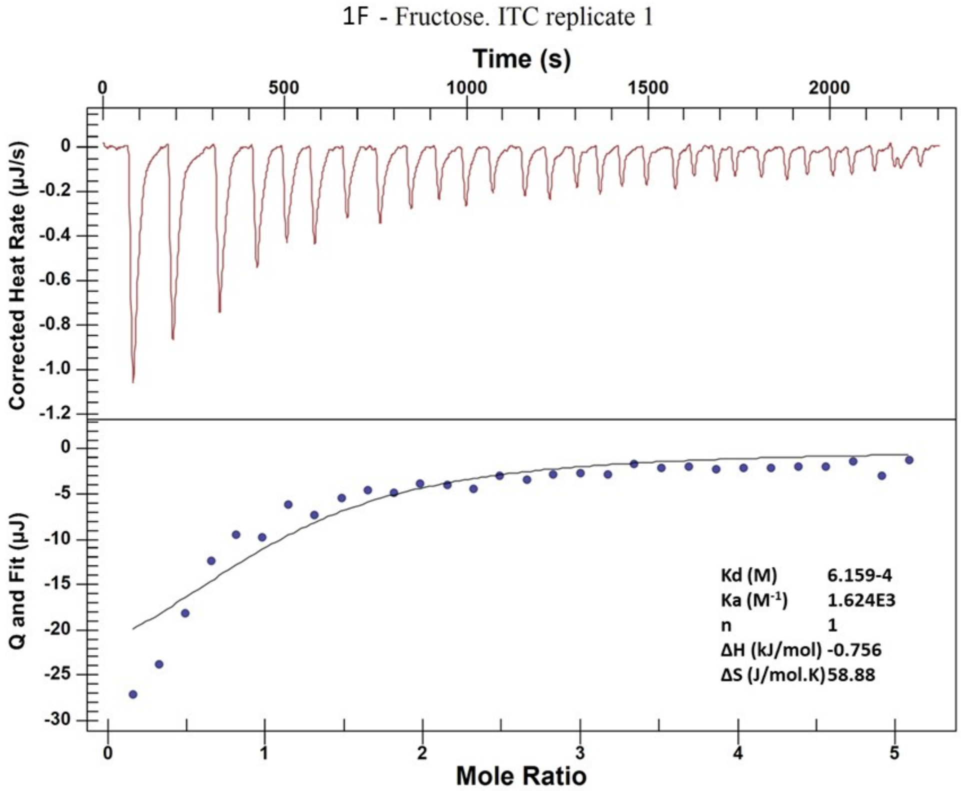

Figure S26: ITC titration of **1F** and fructose.

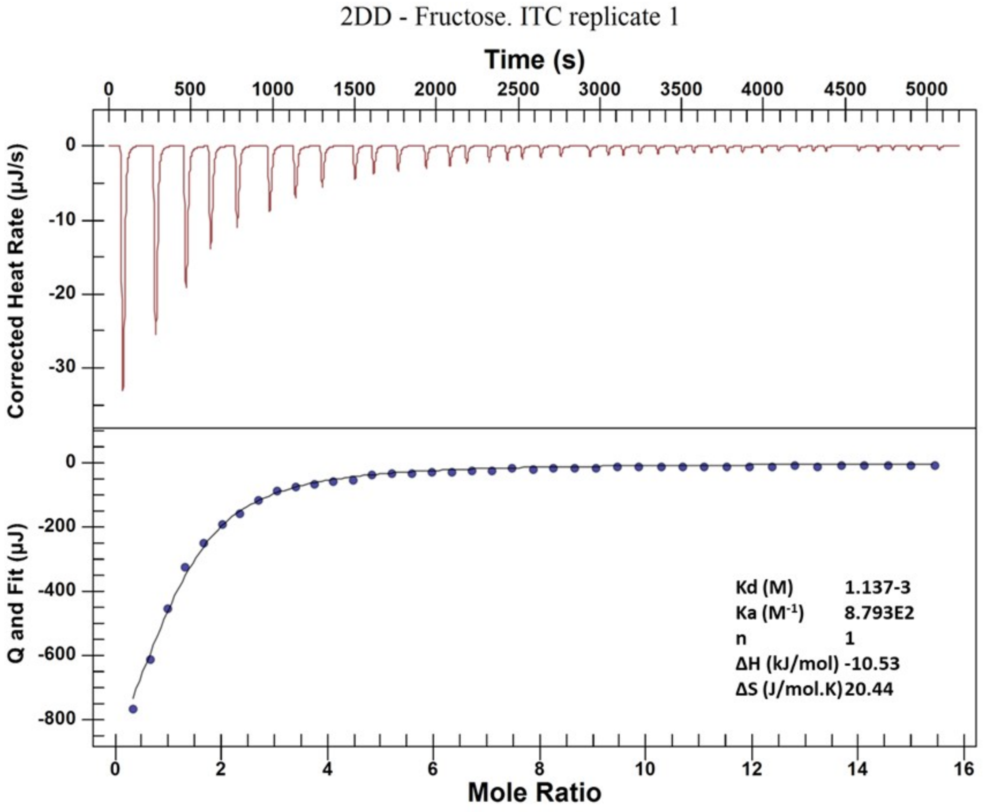

Figure S27: ITC titration of **2DD** and fructose.

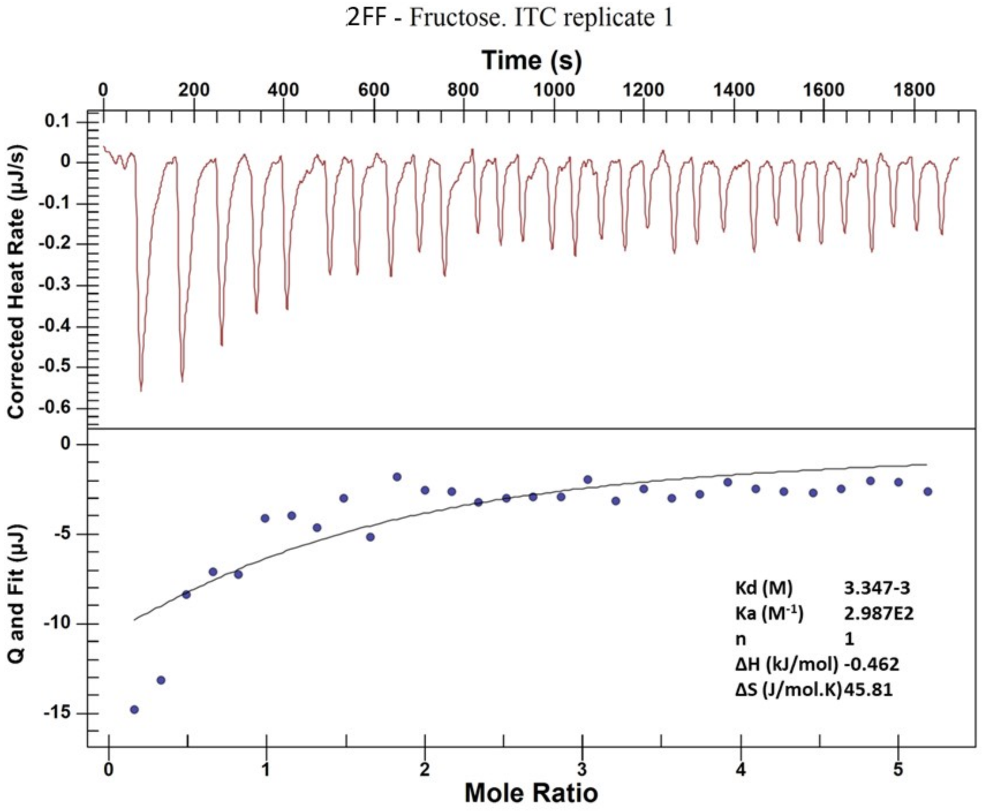

Figure S28: ITC titration of **2FF** and fructose.

Molecular modelling studies

All the molecular models were performed in Maestro version 13.6.121, MMshare Version 6.2.121, Release 2023-2, from Schrödinger. Structures for the complexes between **1F** and fructose were built with **1F** as its monoanionic form (free amine and deprotonated carboxylate) and selected isomers of the saccharide. D-fructose exists as a mixture of five isomers in aqueous solution: three tautomers (linear keto and cyclic five/six-member rings), and two anomers (α/β forms) of each corresponding cyclic hemiacetal. The relative distribution of the different isomers has been thoroughly reported in literature under different experimental conditions, with consistent values of β-D-fructopyranose (65-70%), β-D-fructofuranose (22-25%), α-D-fructofuranose (5-6.5%), α-D-fructopyranose (0.5-3%) and linear ketose (<1%) as the species present in equilibrium in water.[i] Since we did not observe important changes in the distribution of the species by ^{1}H NMR upon addition of **1F**, we focused the modelling studies on the four cyclic isomers (accounting for >99% species). The complexes were submitted to a conformational searches with the MCMM/LMCS sampling approach that combines Monte Carlo conformational searches with low mode sampling.[ii] The generated geometries were optimized with the OPLSE4 force field[iii] in implicit water.[iv] This protocol produced over 250 local minima for each complex. These minima were energetically ordered and selected representative structures with lowest energy were further optimized by Jaguar package in Maestro suite using DFT calculations at the B3LYP-D3[v] level of theory with a 6-31G** basis set. Both gas-phase and C-PCM[vi] solvation model (water) were used in order to obtain a theoretical estimation of the solvation stabilization energy. The same procedure was also applied to free monoanionic **1F**, β-D-fructopyranose, β-D-fructofuranose, α-D-fructofuranose and α-D-fructopyranose to estimate the corresponding binding energies.

1F/β-D-fructopyranose complexes

In this case three representative complexes were identified as depicted in Figure S29 and the relative energies are shown in Table S2.

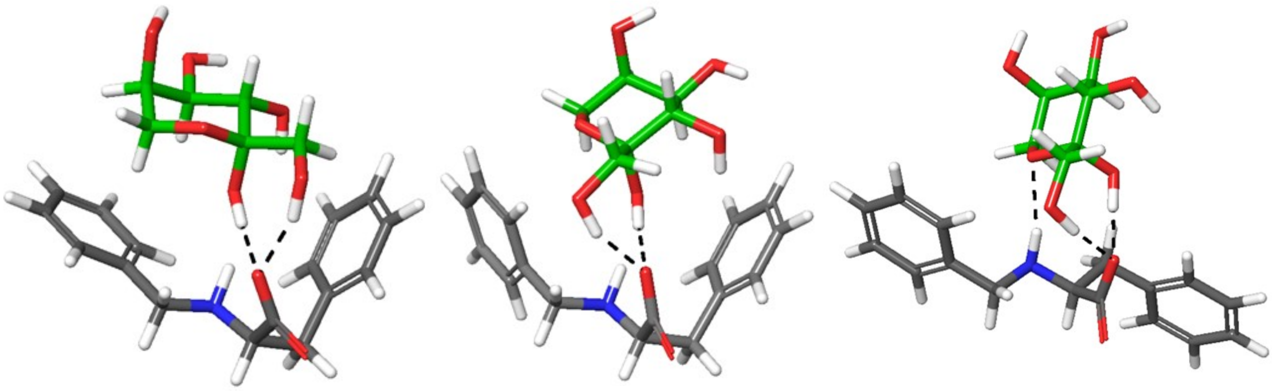

Figure S29: DFT optimized **1F**/β-D-frutopyranose complex1 (left), complex2 (middle) and complex3 (right). The sugar C-atoms are highlighted in green and possible H-bonds are shown in dashed lines.

Table S2: Gas-phase and aqueous solution energies, alongside the theoretical solvation stabilization energy for complexes 1, 2 and 3 formed between receptor 1F and saccharide β-fructopyranose (as depicted in Figure S29). The differences in energies between complex 1 and 2, as well as in complex 1 and complex 3 are also shown.

Complex	sugar	gas-phase E (kcal/mol)	solution E (kcal/mol)	solvation E (kcal/mol)
1F_complex1	β-fructopyranose	-948731.5176	-948804.4184	-72.90078853
1F_complex2	β-fructopyranose	-948749.9888	-948802.8277	-52.83889012
1F_complex3	β-fructopyranose	-948746.087	-948800.1489	-54.06189346
Diff. Comp.(1-2)		18.47117831	**-1.590720105**	-20.06189841
Diff. Comp.(1-3)		14.56936465	**-4.269530412**	-18.83889507

The three complexes are characterized by stabilizing double H-bonds between the **1F**-carboxylate and the hemiacetal anomeric and C1 hydroxyl groups (dashed lines in Figure S29). The two most stable complexes (complex1 and complex2) are very similar in their geometry. In both, the **1F** receptor shows a V-shaped conformation with the two aromatic rings (Phe side chain and benzyl residue) pointing towards the sugar, thus defining, along with the carboxylate group, a tweezer for the saccharide. Complex3 is an open structure stabilized exclusively by H-bonds. By looking to the corresponding energies (both in gas phase and in water), remarkable conclusions can be drawn. First, the extra-stabilization of complex1 is mainly due to the effect of water, which allows us to hypothesize that the tweezer conformation is promoted by the corresponding C-H···π interactions, that are favoured in aqueous media. Besides, complex1 shows a more efficient C-H···π contact, explaining the reversed stability in comparison with complex2 in gas phase versus solution.

1F/β-D-fructofuranose complexes

Two complexes were optimized at DFT level (see Figure S30) named as complex1 (left) and complex2 (right). Their structures are shown below and the absolute and relative energies of these two complexes are shown in Table S3.

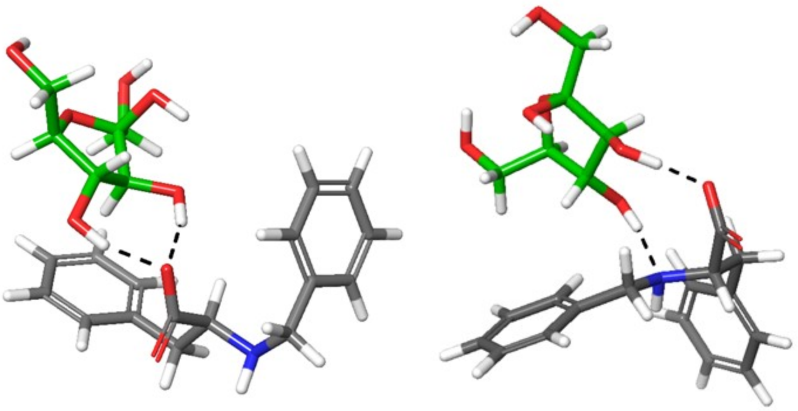

Figure S30: DFT optimized 1F/β-D-frutofuranose complex1 (left) and complex2 (right). The sugar C-atoms are highlighted in green and possible H-bonds are shown in dashed lines.

Table S3: Gas-phase and aqueous solution energies, alongside the theoretical solvation stabilization energy for complexes 1 and 2 formed between receptor 1F and saccharide β-fructofuranose (as depicted in Figure S30). The differences in energies between complex 1 and 2 are also shown.

Complex	sugar	gas-phase E (kcal/mol)	solution E (kcal/mol)	solvation E (kcal/mol)
1F_complex1	β-fructofuranose	-948724.2894	-948795.5299	-71.24041559
1F_complex2	β-fructofuranose	-948739.6501	-948793.3173	-53.66719407
Diff. Comp. (1-2)		15.36064594	**-2.212575578**	-17.57322152

The results show that complex1 is more stable than complex2 by 2.2 kcal/mol. Also data shows that this extra stabilization sources from the effect of the solvent (compare gas-phase vs. solution energies). The binding in both cases mainly occurs through H-bonding while in complex1 also a sugar C-H···π interaction can be proposed with the side chain of Phe amino acid. This extra-stabilization would explain the solvation effect, since it 'hides' the hydrophobic moiety of the sugar from water. Complex2 is more open and no hydrophobic interactions are present.

Complexes with the minor α-isomers

The corresponding complexes formed between **1F** and either α-D-fructofuranose and α-D-fructopyranose were also optimized following the same protocol as previously described. The obtained structures and energies are shown in Figure S31.

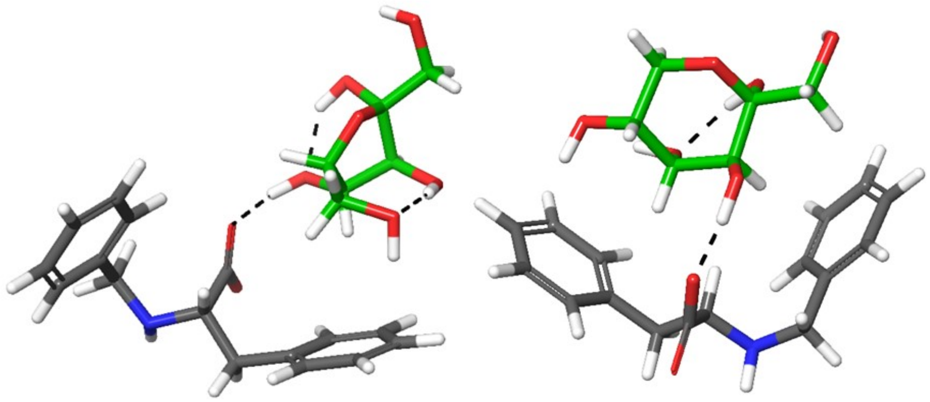

Figure S31: DFT optimized 1F/α-D-frutofuranose (left) and 1F/α-D-frutopyranose (right). The sugar C-atoms are highlighted in green and possible H-bonds are shown in dashed lines.

Table S4: Gas-phase and aqueous solution energies, alongside the theoretical solvation stabilization energy for the complexes 1F with α-fructofuranose and 1F with α-fructopyranose (as depicted in Figure S31).

Receptor	sugar	gas-phase E (kcal/mol)	solution E (kcal/mol)	solvation E (kcal/mol)
1F	α-fructofuranose	-948742.6803	-948794.8114	-52.13106673
1F	α-fructopyranose	-948740.3039	-948796.783	-56.47903502

Comparison of the two major complex species: 1F/β-D-fructopyranose vs. 1F/β-D-fructofuranose

The relative energies for the complexes formed with the two main isomers of fructose in aqueous solution (Table S5) shows that the complex with β-D-fructopyranose is favoured over that with β-D-fructofuranose, explaining why the presence of **1F** does not dramatically modify the isomers proportion.

Table S5: Substraction of the values of gas-phase energy, aqueous solution energy and theoretical solvation stabilization energy for the complexes 1F/β-D-fructopyranose minus 1F/β-D-fructofuranose.

Receptor	Sugar	gas-phase E (kcal/mol)	solution E (kcal/mol)	solvation E (kcal/mol)
1F	β-pyranose-furanose	-7.228207057	**-8.888579995**	**-1.660372938**

Estimation of the binding energies

As a simple estimation of the binding energies for the optimized complexes, we additionally optimized the geometries of the corresponding components, using the same computational protocol. The obtained minima are shown below in Figure S32.

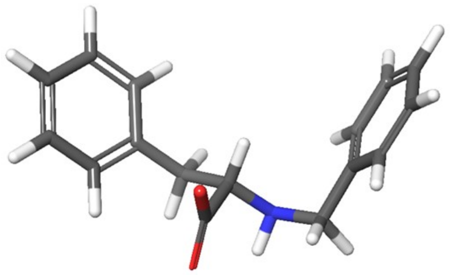

1F

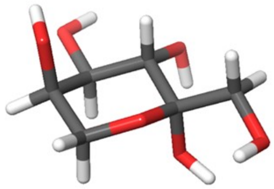

β-D-fructopyranose

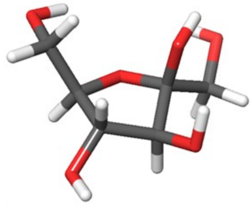

β-D-fructofuranose

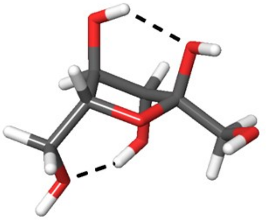

α-D-fructofuranose

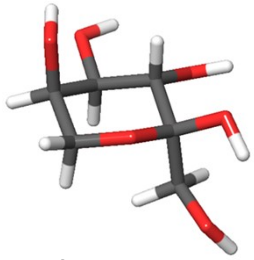

α-D-fructopyranose

Figure S32: DFT optimized geometries for the 1F receptor and fructose isomers.

Table S6 contains the corresponding energies for all the components, **1F** and the four main isomers of D-fructose.

Table S6: Gas-phase and aqueous solution energies, alongside the theoretical solvation stabilization energy for the four main isomers of D-fructose as well as for 1F free (Figure S32).

structure	gas-phase E (kcal/mol)	solution E (kcal/mol)	solvation E (kcal/mol)
β-fructopyranose	-431227.869	-431239.2306	-11.36156932
β-fructofuranose	-431235.9173	-431245.4943	-9.576950786
α-fructofuranose	-431231.4282	-431242.7766	-11.34839176
α-fructopyranose	-431226.5964	-431239.5506	-12.95417193
1F_free	-517468.3403	-517528.7381	-60.39779125

The binding energies were then estimated as:

$$\Delta E_{bind} = E_{complex} - (E_{\mathbf{1F}} + E_{sugar})$$

For each complex, both in the gas phase and in water. The results are shown in Table S7.

Table S7: Estimated binding energies for the complexes formed between 1F and the four main isomers of D-fructose.

Complex	gas-phase E (kcal/mol)	solution E (kcal/mol)	solvation E (kcal/mol)
1F/β-fructopyranose	-27.25998533	**-30.18603181**	-2.926046489
1F/β-fructofuranose	-28.08013175	**-27.56118677**	0.518944981
1F/α-fructofuranose	-42.91179265	**-23.29667638**	19.61511628
1F/α-fructopyranose	-45.36721189	**-28.49428373**	16.87292817

The analysis of the estimated binding energies confirms the spontaneous formation of the complexes between **1F** and the four isomers of fructose. The solvation effect specifically favours the **1F**/β-D-fructopyranose complex, which is the most favoured and the most stable one. This result correlates with the experimental observation that addition of **1F** over a solution of fructose in water does not change the NMR spectra of fructose, suggesting no variation in isomeric composition upon binding.

We can propose that the flexible structure of **1F** allows to dynamically bind all the isomers of the sugar, for which a conventional receptor design would have been difficult. Moreover, in the two most-stable global minima for the two major tautomers, the aromatic rings of the Phe side chain (β-D-fructofuranose) or both side chain and benzyl residue (β-D-fructopyranose) play a key role in the interaction. This would explain the selection of **1F** from the dynamic combinatorial mixture. On the other hand, the folded conformation of **1F** (specially for β-D-fructopyranose) suggests that the receptor conformationally adapts to the substrate, leading to a compact structure that efficiently de-solvates **1F** and, specially, the sugar. De-solvation of host and guest would account for a favourable entropy of binding as experimentally measured by ITC. Finally, the concerted H-bonding of carboxylate of **1F** with anomeric OH and C1-OH can only be proposed with the ketose six-member ring, thus suggesting an explanation for the strong binding to fructose vs. the other hexoses, for which such binding motif is not possible.

---

[i] Barclay, T.; Ginic-Markovic, M.; Johnston, M. R.; Cooper, P.; Petrovsky, N. Observation of the keto tautomer of D-fructose in D2O using 1H NMR spectroscopy. *Carbohydr. Res.* **2012**, *347*, 136-141 https://doi.org/10.1016/j.carres.2011.11.003 and references therein.

[ii] (a) Kolossváry, I.; Guida, W. C. Low-Mode Conformational Search Elucidated: Application to C39H80 and Flexible Docking of 9-Deazaguanine Inhibitors into PNP. *J. Comput. Chem.* **1999**, *20* (15), 1671–1684 https://doi.org/10.1002/(SICI)1096-987X(19991130)20:15<1671::AID-JCC7>3.0.CO;2-Y (b) Kolossváry, I.; Guida, W. C. Low Mode Search. An Efficient, Automated Computational Method for Conformational Analysis: Application to Cyclic and Acyclic Alkanes and Cyclic Peptides. *J. Am. Chem. Soc.* **1996**, *118* (21), 5011–5019 https://doi.org/10.1021/ja952478m (c) Goodman, J. M.; Still, W. C. An Unbounded Systematic Search of Conformational Space. *J. Comput. Chem.* **1991**, *12* (9), 1110–1117 https://doi.org/https://doi.org/10.1002/jcc.540120908

[iii] (a) OPLS4, Schrödinger, Inc., New York, NY, 2021; (b) Lu, C.; Wu, C.; Ghoreishi, D.; Chen, W.; Wang, L.; Damm,

W.; Ross, G. A.; Dahlgren, M. K.; Russell, E.; Von Bargen, C. D.; Abel, R.; Friesner. R. A.; Harder, E.; OPLS4: Improving Force Field Accuracy on Challenging Regimes of Chemical Space. *J. Chem. Theory Comput.* **2021**, *17*, 4291. https://doi.org/10.1021/acs.jctc.1c00302
[iv] Still, W. C.; Tempczyk, A.; Hawley, R. C.; Hendrickson, T. Semianalytical Treatment of Solvation for Molecular Mechanics and Dynamics. *J. Am. Chem. Soc.* **1990**, *112*, 6127–6129. https://doi.org/10.1021/ja00172a038
[v] Grimme, S.; Antony, J.; Ehrlich, S.; Krieg; H. A consistent and accurate *ab initio* parametrization of density functional dispersion correction (DFT-D) for the 94 elements H-Pu. *J. Chem. Phys.* **2010**, 132 (15): 154104. https://doi.org/10.1063/1.3382344
[vi] Cossi, M.; Rega, N.; Scalmani, G.; Barone, V. Energies, structures, and electronic properties of molecules in solution with the C-PCM solvation model. *J. Comput. Chem.* **2003**, *24*, 669-681, https://doi.org/10.1002/jcc.10189
